# UAV Path Planning: A Dual-Population Cooperative Honey Badger Algorithm for Staged Fusion of Multiple Differential Evolutionary Strategies

**DOI:** 10.3390/biomimetics10030168

**Published:** 2025-03-10

**Authors:** Xiaojie Tang, Chengfen Jia, Zhengyang He

**Affiliations:** School of Mechanical Engineering, Sichuan University Jinjiang College, Meishan 620860, China; jiachengfen@scujj.edu.cn (C.J.); hezhengyang@scujj.edu.cn (Z.H.)

**Keywords:** UAV path planning, heuristic algorithm, latin hypercube sampling, dual-population mutation method, DE mutation operator, stochastic perturbation strategy

## Abstract

To address the challenges of low optimization efficiency and premature convergence in existing algorithms for unmanned aerial vehicle (UAV) 3D path planning under complex operational constraints, this study proposes an enhanced honey badger algorithm (LRMHBA). First, a three-dimensional terrain model incorporating threat sources and UAV constraints is constructed to reflect the actual operational environment. Second, LRMHBA improves global search efficiency by optimizing the initial population distribution through the integration of Latin hypercube sampling and an elite population strategy. Subsequently, a stochastic perturbation mechanism is introduced to facilitate the escape from local optima. Furthermore, to adapt to the evolving exploration requirements during the optimization process, LRMHBA employs a differential mutation strategy tailored to populations with different fitness values, utilizing elite individuals from the initialization stage to guide the mutation process. This design forms a two-population cooperative mechanism that enhances the balance between exploration and exploitation, thereby improving convergence accuracy. Experimental evaluations on the CEC2017 benchmark suite demonstrate the superiority of LRMHBA over 11 comparison algorithms. In the UAV 3D path planning task, LRMHBA consistently generated the shortest average path across three obstacle simulation scenarios of varying complexity, achieving the highest rank in the Friedman test.

## 1. Introduction

With the widespread application of unmanned aerial vehicles (UAVs) in complex terrain exploration, disaster response, military reconnaissance, and other fields, three-dimensional path planning has emerged as a core challenge in autonomous navigation systems. The objective is to determine an optimal flight path that minimizes cost while ensuring the UAV navigates through three-dimensional space without violating operational constraints or colliding with obstacles [1,2].

Corresponding to such a complex constraint optimization problem as path planning, scholars have proposed some algorithms and improvement studies. Traditional path planning methods include the A-star algorithm [3], Artificial Potential Fields (APF) [4], and RRT* [5], etc. These algorithms appeared earlier, developed more maturely, and were relatively simple to implement, but for path planning in complex environments, problems such as slow convergence speed would occur. In recent years, swarm intelligence algorithms have become an important method to solve the path planning problem, and this meta-heuristic algorithm simulates the information sharing and mutual learning between biological individuals in nature, with stronger self-learning, self-adaptation, and self-organization [6,7,8,9]. Some algorithms with superior performance are widely used in UAV path planning, including Particle Swarm Algorithm (PSO) [10], Artificial Bee Colony Algorithm (ABC) [11], Whale Optimization Algorithm (WOA) [12], Harris Hawks Optimization (HHO) [13], and Sparrow Search Algorithm (SSA) [14], Dung Beetle Optimizer (DBO) [15], Crested Porcupine Optimizer (CPO) [16] and so on. Li et al. [17] introduced the FP-GPSO algorithm, which uses Fermat points for grouped particle swarm optimization to address the path-planning challenge of composite unmanned aerial vehicles. Experimental findings demonstrated the effectiveness of FP-GPSO in optimizing UAV path planning. Han et al. [18] developed a multi-strategy evolutionary database to enhance the conventional artificial bee colony algorithm, leveraging their brain-inspired evolutionary learning framework to improve cognitive abilities, and simulation results show that the algorithm produces UAV trajectories with better fuel economy and higher safety. Dai et al. [19] proposed a novel whale optimization algorithm (NWOA), which improves the convergence speed and avoids local optima by using adaptive techniques and setting virtual obstacles and introduces improved potential field factors to enhance the dynamic obstacle avoidance ability of mobile robots. Tang et al. [20] improved the Harris Hawk algorithm using several strategies such as dimensional learning-based hunting (DLH) search and applied it to robot path planning, and the results demonstrated the algorithm’s clear advantage in path planning performance. Cheng et al. [21] developed an improved SSA applied to UAV path planning based on the theory of uniform experimental design and obtained good experimental results. Lian et al. [22] added exponentially decreasing inertia weights and adaptive Cauchy mutation strategies to the dung beetle optimizer to solve complex UAV path planning problems in complex 3D environments. Liu et al. [23] proposed a periodic retreat strategy and a visual-auditory synergistic defense mechanism to improve the Crested Porcupine Optimizer (ICPO) for better obstacle avoidance and reduced energy consumption of UAVs.

The Honey Badger Algorithm (HBA) [24] is a nature-inspired optimization algorithm proposed by Hashim et al. in 2021. It is designed to identify optimal solutions by mimicking the intelligent foraging behavior of the honey badger. HBA is characterized by its strong optimization capability, robustness, and simplicity of implementation. Numerous researchers have explored and applied HBA across various fields, yielding promising experimental results, as summarized in Table 1.

Currently, research on the application of the Honey Badger Algorithm (HBA) in UAV path planning remains limited. Hu et al. [33] proposed an improved variant of HBA, named SaCHBA_PDN, which incorporates a Bernoulli shift map, segment optimal decreasing neighborhoods, and adaptive level crossing for path planning. While the algorithm demonstrates strong performance in two-dimensional path planning, it does not address three-dimensional path planning. Therefore, this study explores the application of HBA to tackle the NP-hard problem of UAV 3D path planning [34].

According to the “No Free Lunch (NFL)” theorem [35], no single algorithm can effectively solve all optimization problems. To enhance the applicability of the Honey Badger Algorithm (HBA) for UAV path planning and address its limitations, such as slow convergence speed, an imbalance between exploration and exploitation, and susceptibility to local optima [36,37,38], this study proposes an improved variant, named LRMHBA.

First, Latin hypercube sampling combined with an elite strategy [39] is employed during initialization to ensure a more uniform population distribution and enhance global search efficiency. Second, a stochastic perturbation strategy inspired by the Whale Optimization Algorithm is integrated to improve the algorithm’s ability to escape local optima. Finally, a staged dual-population co-evolutionary strategy incorporating multiple differential evolution variants is introduced. The population is divided into two subpopulations based on their adaptive capabilities. Considering the dynamic trade-off between exploration and exploitation during optimization, different differential evolution variants are assigned to the two subpopulations at various evolutionary stages. Additionally, the elite population obtained during initialization is leveraged to guide the differential mutation process, improving the balance between exploration and exploitation and enhancing the algorithm’s global optimization capability.

The following are this paper’s primary contributions:

- Effective fusion of the randomized perturbation strategy in the whale algorithm and the honey badger algorithm.
- Proposing a staged two-population coevolutionary strategy that incorporates multiple differential variation approaches.
- Proposing an improved HBA algorithm (LRMHBA) that combines Latin hypercubic sampling with an elite strategy, a randomized perturbation strategy, and a staged two-population co-evolutionary strategy that fuses multiple differential variability approaches.
- Comparative performance tests were conducted on the LRMHBA algorithm against various competing algorithms, including highly referenced algorithms and their variants, recently developed high-performance algorithms, the champion algorithm, as well as the original HBA and its variant, using the CEC2017 test suite, with evaluations covering both low-dimensional (30-dimensional) and high-dimensional (100-dimensional) function optimization. Statistical analyses, including the Wilcoxon rank-sum test and Friedman test, along with ablation and exploration-exploitation experiments, were performed to validate the advancements of LRMHBA.
- The UAV flight cost is defined and three UAV 3D simulation scenarios from simple to complex are established, and the performance of path planning for each scenario is compared and analyzed with the LRMHBA algorithm and other competing algorithms, and the outcomes demonstrate the superiority of the LRMHBA method in the UAV path planning problem as well.

The paper is organized as follows: Section 2 describes the UAV path planning modeling approach. Section 3 describes the principles of the HBA algorithm. Section 4 describes specific improvements to the HBA algorithm. Section 5 is a discussion of testing and analyzing the LRMHBA algorithm using the CEC2017 test suite. Section 6 is the UAV path planning simulation test analysis. Section 7 summarizes the entire thesis research work.

## 2. UAV Path Planning Modeling

### 2.1. Environmental Modeling

Environmental modeling refers to the process of converting physical environmental information into digital models that can be processed by computer algorithms, serving as the prerequisite and foundation for UAV path planning.

#### 2.1.1. Base Terrain Model

We adopted a commonly used functional simulation method to generate realistic terrain patterns [40], as expressed in Equation (1):

(1) $$\;\;z_{1}\left(x,y\right)=k\cdot (\sin\left(y+a\right)+b\cdot sinx+c\cdot cos\left(d\cdot \sqrt{x^{2}+y^{2}}\right)+e\cdot cosy+f\cdot sin(g\cdot \sqrt{x^{2}+y^{2}})$$

where $z_{1}$ is the height value at coordinate (x,y); k, a, b, *c*, d, e, f, g are the scaling coefficients controlling digital terrain characteristics.

#### 2.1.2. Mountain Model

Natural mountainous terrain poses the most significant threat to UAV navigation. We employ an exponential superposition function to characterize such features [41], as defined in Equation (2):

(2) $$\;z_{2}\left(x,y\right)=\sum \left(h_{i}e^{-\frac{{\left(x-x_{i}\right)}^{2}}{a_{i}^{2}}-\frac{{\left(y-y_{i}\right)}^{2}}{b_{i}^{2}}}\right)$$

where $z_{2}$ is the height value at the point (*x*, *y*); $h_{i}$ is the peak altitude of the ith mountain; $\left(x_{i},y_{i}\right)$ are the centroid coordinates of the ith mountain; $a_{i}$ and $b_{i}$ are the slope parameters in x/y directions for the ith mountain.

### 2.2. Operational Constraints

The proposed path planning model simulates UAV operations in both simple and complex hazardous environments, incorporating multiple constraint costs [42,43] to enhance the practical relevance and applicability of the study.

#### 2.2.1. Flight Distance Cost

Limited by fuel capacity and consumption rate, UAVs operate under strict flight range constraints. The distance cost function balancing flight efficiency and obstacle avoidance is formulated as follows:

(3) $$\;C_{L}=\sum_{i=1}^{n-1}\sqrt{({x_{i+1}-x_{i})}^{2}+{\left(y_{i+1}-y_{i}\right)}^{2}+{\left(z_{i+1}-z_{i}\right)}^{2}}$$

where ($x_{i}$, $y_{i}$, $z_{i}$) denotes the 3D coordinates of the ith waypoint; n is the total number of waypoints.

#### 2.2.2. Flight Altitude Cost

Maintaining appropriate flight altitude enhances fuel efficiency and operational safety. To ensure stealth performance, UAVs require stable low-altitude flight patterns. The altitude deviation cost is defined in Equation (4) with its mean reference in Equation (5):

(4) $$C_{H}=\sum_{i=1}^{n}\sqrt{{\left(z_{i}-\bar{z}\right)}^{2}}$$

(5) $$\bar{z}=\frac{1}{n}\cdot \sum_{i=1}^{n}z_{i}$$

where $z_{i}$ is the altitude at the ith waypoint; $\bar{z}$ is the mean flight altitude.

#### 2.2.3. Turning Maneuver Cost

Sharp turns jeopardize UAV stability and controllability. The turning angle is usually not greater than the pre-set maximum turning angle. The cost function for flight turning is presented as follows:

(6) $$\;\;C_{C}=\sum_{i=1}^{n-2}\left(\cos\emptyset -\cos\left(\theta_{i}\right)\right)$$

(7) $$\cos\left(\theta_{i}\right)=\frac{b_{i}^{T}\cdot b_{i+1}}{\left|b_{i}\right|\cdot \left\lceil b_{i+1}\right\rceil }$$

where $\theta_{i}$ denotes the turning angle between adjacent path segments; Φ is the maximum allowable turning angle; $b_{i}$ is the direction vector of the ith path segment.

#### 2.2.4. Terrain Clearance Constraint

To prevent terrain collisions during mission execution, planned trajectories must maintain altitude superiority over terrain with designated safety margins. The terrain clearance cost is formulated as follows:

(8) $$C_{M}=\sum_{i=1}^{n}f\left(d_{i}\right)$$

(9) $$\;\;f\left(d_{i}\right)=\left\{\begin{array}{c} \begin{array}{cc} {\left(1-\frac{d_{i}}{2SD}\right)}^{2}, & if\;d_{i}<2SD \end{array}\\ \begin{array}{cc} 0, & \;\;\;\;\;\;\;\;\;\;\;\;\;\;\;\;otherwise \end{array} \end{array}\right.$$

(10) $$d_{i}=z_{i}-h_{i}$$

where $z_{i}$ denotes the terrain elevation at the ith waypoint and *SD* denote the Mandatory safety clearance (0.2 km in this study).

#### 2.2.5. Obstacle Threat Cost

UAVs must avoid collisions with obstacles and radar threat zones modeled as vertical cylinders where flight operations are strictly prohibited at all altitudes. The threat cost function enforces safety buffers around threat sources, as defined in Equations (11)–(13):

(11) $$C_{T}=\sum_{i=1}^{n}\sum_{k=1}^{m}f\left(d_{ik}\right)$$

(12) $$f\left(d_{ik}\right)=\left\{\begin{array}{c} \begin{array}{cc} {\left(1-\frac{d_{ik}}{{2R}_{k}\cdot SF}\right)}^{2}, & if\;d_{ik}<{2R}_{k}\cdot SF \end{array}\\ \begin{array}{cc} \;\;\;0, & \;\;\;\;\;\;\;\;\;\;\;\;\;\;\;\;\;\;\;\;\;\;\;\;\;\;\;otherwise \end{array} \end{array}\right.$$

(13) $$d_{ik}=\sqrt{{\left(x_{i}-x_{k}\right)}^{2}+{\left(y_{i}-y_{k}\right)}^{2}}$$

where $\left(x_{k},y_{k}\right)$ denotes the centroid coordinates of the kth threat zone; $R_{k}$ denotes the radius of the kth cylindrical threat zone; SF is the safety factor for threat zones (1.2 in this study).

**Figure 1:**
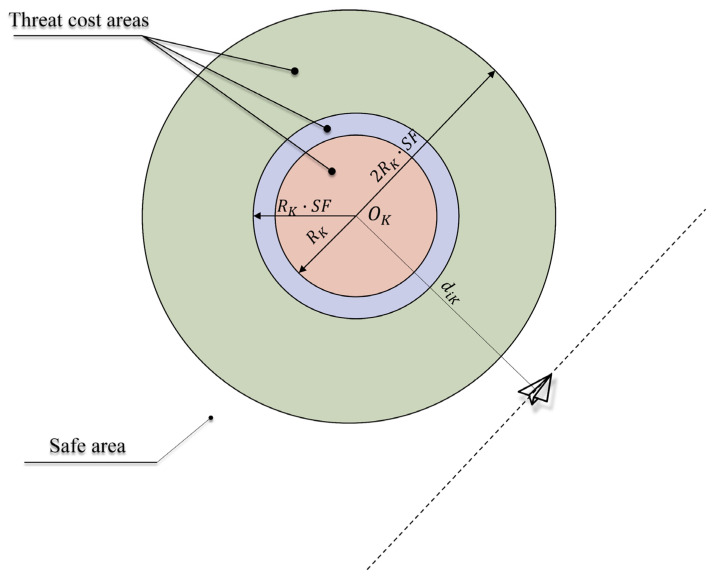
Schematic of the threat cost model.

As illustrated in Figure 1, $O_{k}$ denotes the centroid of the kth threat source. The penalty activates when $d_{ik}<{2R}_{k}\cdot SF$, with threat cost increasing quadratically as the distance decreases.

The weighted sum of each individual cost function is the overall cost function, if the proposed path is viable. Otherwise, the total cost function is assigned an excessively high penalty value, which in this paper is set to 10,000:

(14) $$\;C_{total}=\left\{\begin{array}{c} \begin{array}{cc} \mu_{L}C_{L}+\mu_{H}C_{H}+\mu_{C}C_{C}+\mu_{M}C_{M}+\mu_{T}C_{T} & if\;path\;feasible \end{array}\\ \begin{array}{cc} 10,000,\;\;\;\;\;\;\;\;\; & \;\;\;\;\;\;\;\;\;\;\;\;\;\;\;\;\;\;\;\;\;\;\;\;\;\;\;\;\;\;\;\;\;\;\;\;\;otherwise \end{array} \end{array}\right.$$

where $\mu_{L},\mu_{H},\mu_{C},\mu_{M},\mu_{T}$ are weighting coefficients with ∑μ=1.

## 3. Honey Badger Algorithm (HBA)

This section outlines the principle of the Honey Badger Algorithm (HBA), where the parameters $r_{1}$ to $r_{7}$ in the formula are random numbers between 0 and 1.

### 3.1. Population Initialization

The population is randomly initialized within predefined boundaries using:

(15) $$X_{i}={lb}_{i}+r_{1}\cdot \left(ub_{i}-{lb}_{i}\right)$$

where $X_{i}$ is the position vector of the ith individual; lb and ub denote the lower/upper bounds of search space.

### 3.2. Excavation Phase

Honey badgers perform cardioid-shaped movements during prey excavation, mathematically modeled as:

(16) $$\;\;X_{new}=X_{prey}+Dir\cdot \beta \cdot I\cdot X_{prey}+F\cdot r_{2}\cdot \alpha \cdot d_{i}\cdot \left|\cos\left(2\cdot \pi \cdot r_{3}\right)\times \left(1-\cos\left(2\cdot \pi \cdot r_{4}\right)\right)\right|$$

where $X_{new}$ denotes the updated position of the individual honey badger; $X_{prey}$ denotes the global best position; β is the food acquisition capability factor, β≥1; I is the prey control intensity (Equations (17)–(19)); α is the density factor for exploration-exploitation transition (Equation (20)); Dir is the Search direction operator (Equation (21)).

(17) $$\;I_{i}=\frac{r_{5}\cdot S}{4\cdot \pi \cdot d_{i}^{2}}$$

(18) $$S={\left(X_{i}-X_{i+1}\right)}^{2}$$

(19) $$d_{i}=X_{prey}-X_{i}$$

where S is the source intensity concentration; $d_{i}$ denotes the distance to prey.

(20) $$\;\;\;\alpha =C\cdot \exp\left(\frac{-t}{t_{max}}\right)$$

where $t_{max}$ is the maximum iterations; C is a constant ≥1 (typically C=2).

(21) $$\;\;Dir=\left\{\begin{array}{c} \begin{array}{cc} 1, & if\;r_{6}\leq 0.5 \end{array}\\ \begin{array}{cc} -1,\;\; & \;else\;\;\;\;\;\;\;\; \end{array} \end{array}\right.$$

### 3.3. Honey Harvesting Phase

Simulating honey badgers following honeyguide birds to beehives:

(22) $$\;X_{new}=X_{prey}+Dir\cdot r_{7}\cdot \alpha \cdot d_{i}$$

## 4. LRMHBA Algorithm

### 4.1. Hybrid LHS Initialization and Elite Guidance

Conventional randomized initialization in the honey badger algorithm may result in suboptimal population distribution and diminished diversity, leading to increased stochastic uncertainty during optimization. To address this limitation, we propose a hybrid initialization strategy integrating Latin Hypercube Sampling (LHS), which enhances spatial uniformity and improves global exploration capabilities.

Latin Hypercube Sampling (LHS) [44,45], proposed by McKay et al. in 1979, is a stratified sampling technique for multivariate parameter distributions. Compared with random sampling, LHS demonstrates superior space-filling properties through its uniform stratification mechanism, particularly when handling limited sample sizes. This method ensures comprehensive coverage of the entire parameter space while capturing tail distribution characteristics.

**Figure 2:**
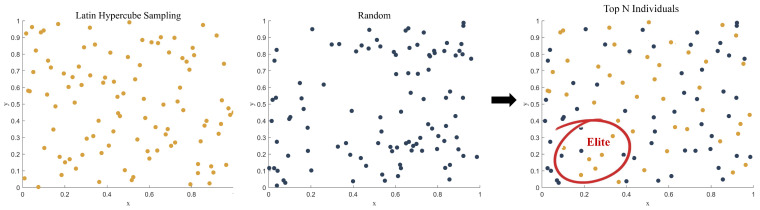
Schematic of hybrid LHS initialization and elite guidance.

To address the uneven initialization issue, we implement a hybrid initialization strategy, as shown in Figure 2:

- Generate N samples using LHS for uniform spatial coverage.
- Create another N sample through random sampling.
- Select the top N individuals by fitness ranking from the combined pool.
- Extract the elite 20% individuals to guide the proposed dual-population framework.

### 4.2. Stochastic Perturbation Strategy

#### Premature Convergence Analysis

In the original HBA method, position updates are primarily influenced by the global best individual $X_{prey}$, which can lead to premature convergence as the population tends to cluster around local optima. To enhance global search capability, we introduce a stochastic perturbation mechanism inspired by the Whale Optimization Algorithm (WOA), regulated by the adaptive coefficient A. When |A|≥1, the search strategy incorporates stochastic perturbation; otherwise, the honey badger’s position is updated based on $X_{prey}$. The coefficient A is calculated as follows:

(23) $$A=2m\cdot r_{8}-m$$

(24) $$\;m=2-\frac{2FES}{MaxFES}$$

where $r_{8}$ is a random number, $r_{8}\in [0,1];$ m linearly decreases from 2 to 0; FES is the current number of function evaluations; MaxFES is the Maximum function evaluation number.

When ∣A∣≥1, the modified position update equation and the redefined distance metric $d_{i}$ are expressed as:

(25) $$X_{new}=X_{rand}+F\cdot \beta \cdot I\cdot X_{rand}+F\cdot r_{2}\cdot \alpha \cdot d_{i}\cdot \left|\cos\left(2\cdot \pi \cdot r_{3}\right)\times \left(1-\cos\left(2\cdot \pi \cdot r_{4}\right)\right)\right|$$

(26) $$X_{new}=X_{rand}+F\cdot r_{7}\cdot \alpha \cdot d_{i}$$

(27) $$\;\;\;d_{i}=X_{rand}-X_{i}$$

The coefficient $\left|A\right|$ exhibits a linear throughout the iteration process. In the initial stage, a high $\left|A\right|$ value activates a random perturbation strategy to prevent premature population aggregation and enhance coverage in the search space. As $\left|A\right|$ decreases during iterations, the algorithm gradually transitions to the precise exploitation of promising regions.

### 4.3. The Staged Dual-Population Co-Evolutionary Strategy Integrating Multiple Differential Evolution Variants

#### 4.3.1. Motivation and Framework

To improve the convergence efficiency and convergence accuracy of the HBA algorithm, we propose a staged dual-population co-evolutionary strategy integrating multiple differential evolution variants, which is set to be executed when the optimal value has not changed in 150 function evaluation times. This approach leverages complementary characteristics of diverse differential evolutionary variants to balance exploration and exploitation dynamically.

#### 4.3.2. DE Mutation Operators

The DE Mutation Operator is one of the core operators of the Differential Evolution (DE) algorithm [46], which serves to generate new candidate solutions (variant individuals) to guide the population to search towards a more optimal solution space. The following canonical DE mutation strategies are adopted due to their proven efficacy [47,48,49,50,51,52]:

(28) $$DE/rand/1:\;\;\;\;\;\;V_{i,G}=X_{r9,G}+F\cdot \left(X_{r10,G}-X_{r11,G,}\right)$$

(29) $$\;DE/rand/2:\;\;\;\;\;\;V_{i,G}=X_{r9,G}+F\cdot \left(X_{r10,G}-X_{r11,G}\right)+F\cdot \left(X_{r12,G}-X_{r13,G}\right)$$

(30) $$DE/best/1:\;\;\;\;\;\;V_{i,G}=X_{best,G}+F\cdot \left(X_{r9,G}-X_{r10,G}\right)$$

(31) $$DE/best/2:\;\;\;\;\;\;V_{i,G}=X_{best,G}+F\cdot \left(X_{r9,G}-X_{r10,G}\right)+F\cdot \left(X_{r11,G}-X_{r12,G}\right)$$

(32) $$DE/current-to-best/1:\;\;\;\;\;\;V_{i}=X_{i,G}+F\cdot \left(X_{best,G}-X_{ri,G}\right)+F\cdot \left(X_{r9,G}-X_{r10,G}\right)$$

where $X_{rk,G}$ denotes the randomly selected individual from generation G; $X_{best,G}$ is the global best individual; $X_{i,G}$ is the ith individual of generation G; $V_{i,G}$ is the mutated individual of generation G; and F is the scaling factor.

Additionally, Layeb, A. et al. [53] (2024) proposed two more differential mutation strategies, which have been validated by extensive experiments:

(33) $$DE/mean-current/2\;\;\;\;\;V_{i,G}=X_{c1,G}+F\cdot \left(X_{c1,G}-X_{i,G}\right)+F\cdot \left(X_{c2,G}-X_{i,G}\right)$$

(34) $$DE/mean-current-best/2\;\;\;\;\;V_{i}=X_{best,G}+F\cdot \left(X_{c1,G}-X_{i,G}\right)+F\cdot \left(X_{c2,G}-X_{i,G}\right)$$

where

(35) $$\;X_{c1,G}=\frac{X_{r9,G}+X_{r10,G}}{2}$$

(36) $$X_{c2,G}=\frac{X_{r9,G}+X_{best,G}}{2}$$

#### 4.3.3. Dual-Population Mutation Method with Elite Individuals

Single mutation strategy fails to address diverse evolutionary demands across population members, because high-fitness individuals (clustered near the current optimum) require intensified exploitation, while low-fitness individuals (distributed in peripheral regions) demand enhanced exploration [54].

To resolve this dichotomy, we implement fitness-based bipartite grouping:

- Group A (Top 50% fitness): Focused on precision exploitation.
- Group B (Bottom 50% fitness): Dedicated to spatial exploration.

At the same time, considering that the population’s search behavior and needs evolve throughout the iteration process, we have designed the following configuration of differential evolution strategies to better address the balance between global exploration and local exploitation. This configuration allows the algorithm to adapt its strategy dynamically throughout the optimization process, thereby finding the optimal balance between global and local optima and ultimately improving the optimization performance.

1. Phase I (Initial 2/3 iterations): Exploration Emphasis.

- Group A: DE/mean-current/2.
- Group B: DE/rand/1.

Group A maintains the exploitation potential and group B breaks through the local optimum through randomized search.

2. Phase II (Final 1/3 iterations): Exploitation Emphasis.

- Group A: DE/current-to-best/2.
- Group B: DE/mean-current/2.

Group A focuses on fine-grained search in optimal neighborhoods, and group B maintains moderate exploration to avoid precocity.

Throughout the process, the elite individuals selected during initialization guide the mutation process in DE/mean-current/2. Consequently, Formula (36) is updated to:

(37) $$\;X_{c2,G}=\frac{X_{r9,G}+X_{elite}}{2}$$

where $X_{elite}$ represents the elite individual obtained during initialization.

After mutation and crossover, the individuals are updated using a greedy strategy:

(38) $$X_{i,G+1}=\left\{\begin{array}{c} \begin{array}{cc} u_{i,G}, & \;\;\;\;\;\;if\;f\left(u_{i,G}\right)\leq f(X_{i,G}) \end{array}\\ \begin{array}{cc} X_{i,G},\; & \;\;\;\;\;\;\;otherwise\;\;\;\;\;\;\;\;\;\;\;\;\;\;\;\;\;\; \end{array} \end{array}\right.$$

where f(·) denotes fitness evaluation.

In summary, the three improvement strategies work together to form an integrated optimization mechanism through phased coordination. Latin hypercube sampling ensures a geometrically homogeneous initialization of the population, with the top 20% of elite individuals guiding subsequent dual-population operations as potential global optima. During iterations, the stochastic perturbation strategy dynamically prevents premature convergence by adaptively adjusting the coefficient. Simultaneously, the staged dual-population co-evolutionary strategy integrates population characteristics, differential evolution mutation operator characteristics, and the evolving demands of the iterative process, gradually enhancing local exploitation while preserving exploration capabilities. This phased collaboration enables LRMHBA to maintain a balanced exploration-exploitation dynamic throughout the entire optimization process.

### 4.4. Pseudocode and Flowchart of LRMHBA

**Figure 3:**
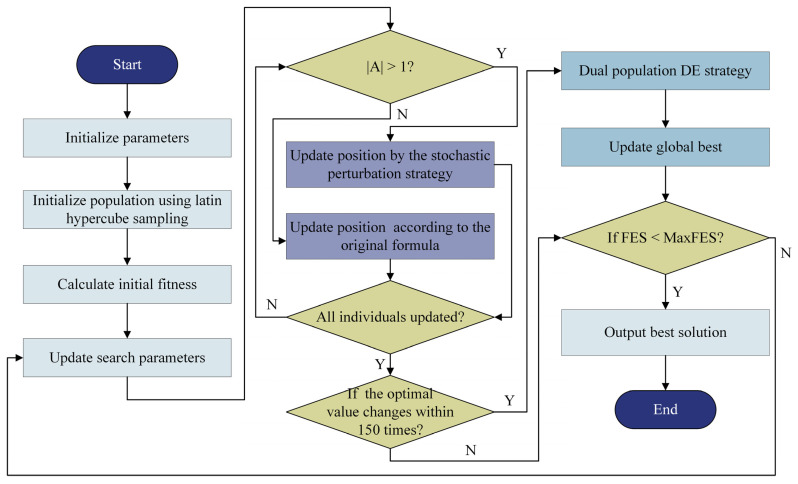
Flowchart of LRMHBA.

Figure 3 displays the flowchart of LRMHBA, which combines the three tactics discussed in this section. Algorithm 1 displays the pseudocode.
**Algorithm 1** Pseudocode of LRMHBA1: Initialize population X using Latin hypercube sampling and elite strategy
2: while FES ≤ Max_FES **do**
3:  Calculate α, I,  m, A using Equations (17), (20), (23) and (24).
4:  for each individual i **do**
5:    if |A|>1 **then**
6:     Update position using random individual using Equations (25) and (26).
7:    **else**
8:    Update position using X_prey using Equations (16) and (22).
9:    **end if**
10:     Update if better solution found.
11:   **end for**
12:    if FES>150 and no improvement in last 150 evaluations **then**
13:   if FES ≤ 2×MaxFES/3 **then**
14:      Set population ratios: 0% mean-current/2, 50% current-to-best/2, 50% rand/1
15:   **else**
16:      Set population ratios: 50% mean-current/2, 50% current-to-best/2, 0% rand/1
17:   **end if**
18:   Sort population by fitness and divide into groups
19:   **for** each group **do**
20:    **if** Group 1 (mean-current/2) **then**
21:       Apply mutation using Equation (33).
22:    **else if** Group 2 (current-to-best/2) **then**
23:       Apply mutation using Equation (32).
24:    **else if** Group 3 (rand/1) **then**
25:       Apply mutation using Equation (28).
26:    **end if**
27:    Apply binomial crossover with probability.
28:    Update if better solution found.
29:   **end for**
30:  **end if**
31:  Update X_prey and Food_Score if better solution found.
32: **end while**
33: Return Food_Score, X_prey.

### 4.5. Time Complexity Analysis

Let N denote the population size, D represent the problem dimension, and MaxFES indicate the maximum number of function evaluations. The original Honey Badger Algorithm (HBA) exhibits a time complexity of $O(MaxFES\cdot \left(N\cdot D\right)).$ When analyzing the computational complexity of the enhanced LRMHBA algorithm relative to HBA, the extra operations and their respective complexities include:

- Latin hypercube sampling during initialization: O(N·D)
- Elite strategy sorting in the initialization phase: O(N·logN)
- Stochastic perturbation strategy: O(1)
- Dual-population mutation method: O(N·log(N⁄150))

Among them, the time complexity of the Latin hypercube sampling and random perturbation strategies is negligible, while the Dual-Population Mutation Method may be triggered only once every 150 times, and its computation time is also approximately negligible with respect to the main loop when the problem size increases, so the total time complexity of the LRMHBA is O(MaxFES·(N·D+N·log(N⁄150))), which is approximately equal to O(MaxFES·(N·D)). So the LRMHBA algorithm basically adds no extra computational cost compared to the baseline HBA algorithm.

## 5. Algorithm Performance Testing and Analysis

This section evaluates the effectiveness of the LRMHBA algorithm using the CEC2017 benchmark suite. The CEC2017 test suite contains 29 functions of various types. It provides a comprehensive evaluation of the LRMHBA algorithm’s performance across different problem types. The dimensions and search ranges of the test functions within the benchmark are detailed in Appendix A.

To analyze the LRMHBA algorithm’s performance in terms of convergence accuracy, convergence speed, and exploration-exploitation capabilities, the following four categories of algorithms were selected for comparison:

- Classical high-citation algorithms and their variants: Particle swarm optimization (PSO), Differential evolution (DE), Whale optimization algorithm (WOA), AOA (Arithmetic optimization algorithm, 2021) [55], GQPSO (Gaussian quantum-behaved particle swarm optimization, 2010) [56];
- Recently proposed algorithms and their variants (within the past two years): PO (Parrot Optimizer, 2024) [57], (Dung Beetle Optimizer (DBO), QHDBO (2024) [58];
- Champion algorithm: LSHADE [59];
- HBA algorithm and its variant: HBA, SaCHBA_PDN.

Every algorithm’s parameters were chosen based on the values suggested by the corresponding sources. as detailed in Table 2. The experimental analysis included performance testing on functions with different dimensions, ablation studies, and exploration-exploitation capability experiments.

The maximum number of function evaluations (FES) was set at 100,000, and the population size for all algorithms was set at 100 to guarantee equity. To reduce experimental randomness and enhance the reliability of results, each algorithm was independently run 30 times. The optimal value, mean value, and standard deviation of the test functions were calculated for both low-dimensional (30 dimensions) and high-dimensional (100 dimensions) settings. The software used for the experiments was Matlab R2021b.

### 5.1. Results Analysis on CEC2017

Table 3 and Table 4 show the experimental results for dimensions 30 and 100, respectively. The best outcomes are indicated in bold for each performance metric (optimal value, mean value, and standard deviation). As shown in Table 3, when the dimension is 30, out of a total of 87 metrics (29 functions × 3 metrics), the LRMHBA algorithm outperformed other competing algorithms in 35 metrics, ranking first. Specifically, for functions CEC09, CEC11, and CEC12, LRMHBA achieved the best results across all three metrics. LSHADE ranked second with 19 best metrics, followed by HBA and SaCHBA_PDN, each with 10 best metrics.

From Table 4, it can be observed that the advantage of LRMHBA becomes even more pronounced when the dimension increases to 100. It ranked first in 44 metrics and achieved better average values than other competing algorithms for 24 functions. These results demonstrate that LRMHBA has a highly competitive global optimization capability and excellent stability.

**Figure 4:**
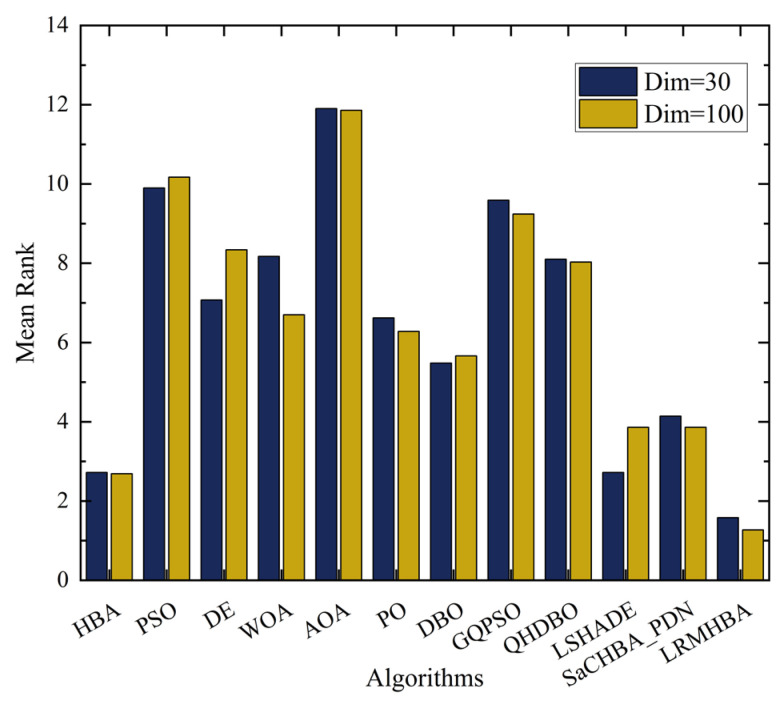
Statistical results of Friedman test rankings in 30 and 100 dimensions.

The final row for each function in Table 3 and Table 4 presents the Friedman test rankings of the algorithms for solving the respective problem. Additionally, the overall mean rank and ranking of the algorithms are provided at the end of the tables. For the 30-dimensional problems, LRMHBA ranked first in 18 functions, whereas for the 100-dimensional problems, it ranked first in 24 functions. Among all the algorithms, LRMHBA achieved the best average rankings, with a rank of 1.58 for 30-dimensional problems and 1.27 for 100-dimensional problems. These results demonstrate that LRMHBA outperforms all other algorithms on the CEC2017 benchmark, with its advantage being particularly pronounced in high-dimensional problems. The summary statistics of the average ranks from the Friedman test for all algorithms at both 30 and 100 dimensions are shown in Figure 4.

**Figure 5:**
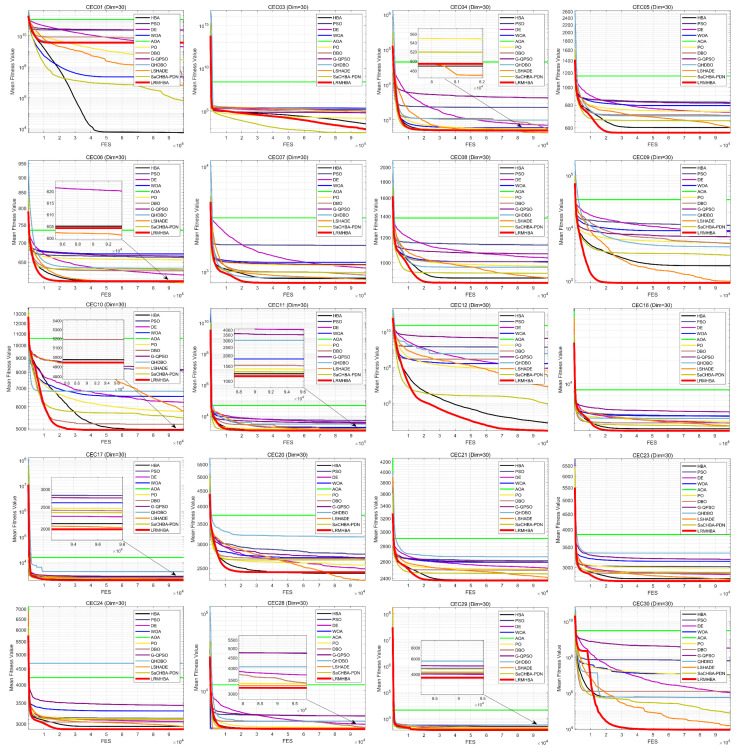
CEC2017 functions iteration curves (dim = 30).

**Figure 6:**
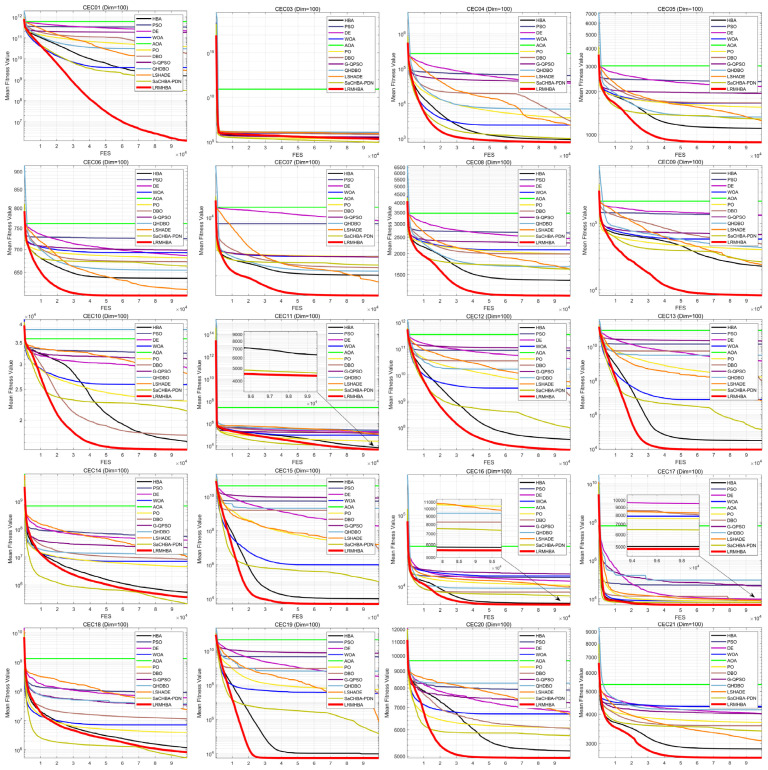

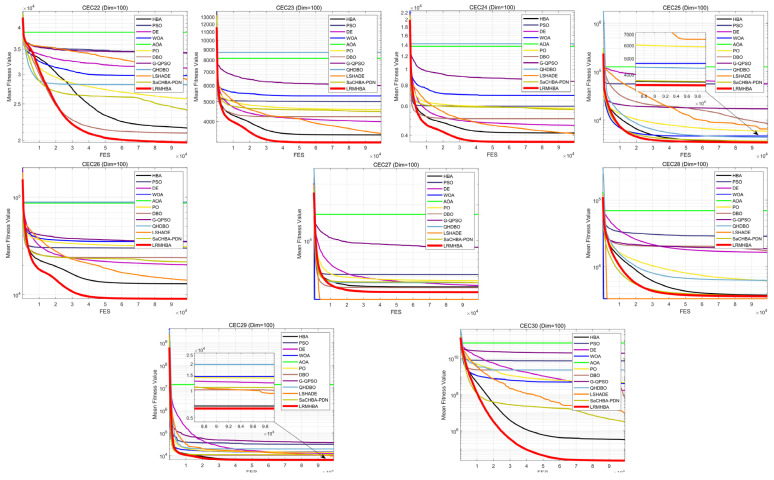
CEC2017 functions iteration curves (dim = 100).

Figure 5 and Figure 6 display the convergence curves for every algorithm that was tested. Due to space limitations, only the convergence curves for 20 functions in the 30-dimensional case are presented. From the figures, it can be observed that LRMHBA achieved better average fitness values on most benchmark functions. Compared to the most competitive algorithms, LSHADE, HBA, and SaCHBA_PDN, LRMHBA generally converged faster than LSHADE and HBA. Although LRMHBA’s early-stage convergence speed was slower than SaCHBA_PDN, the latter was more prone to getting trapped in local optima. In contrast, LRMHBA demonstrated the ability to gradually converge to better values as the number of function evaluations increased, highlighting its superior global optimization capability.

**Figure 7:**
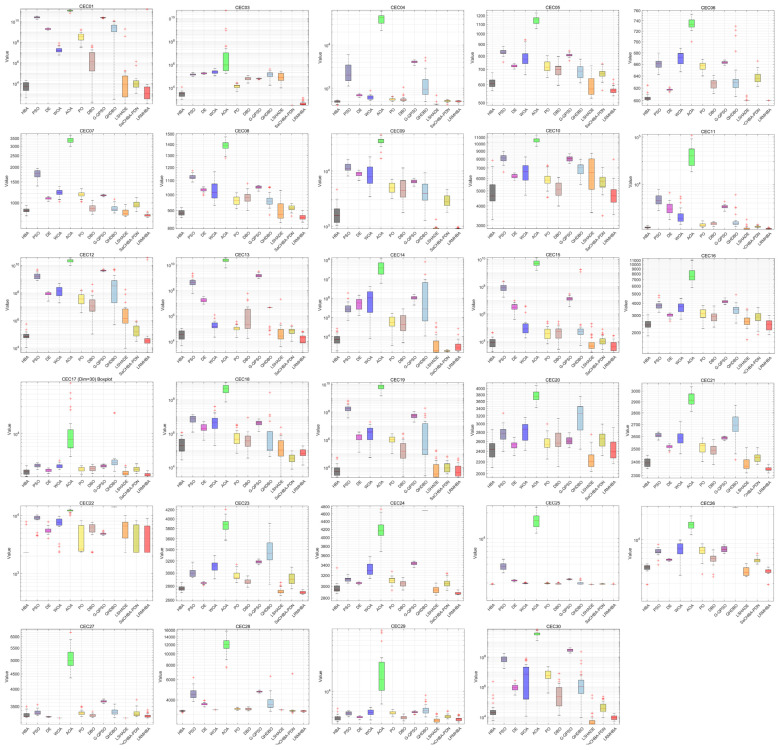
Boxplots of all the algorithms on CEC2017 (Dim = 30).

Figure 7 shows the boxplots of all the algorithms on CEC2017 in 30 dimensions, from which it can be seen that although LRMHBA has a few outliers on some of the test functions, its box lengths are very short, and its median and mean rank first on most of the test functions compared to the other compared algorithms, which indicates that the LRMHBA algorithm has a very good stability.

The statistically significant findings of the Wilcoxon Rank-Sum Test for 100-dimensional issues are shown in Table 5. A *p*-value of less than 0.05 indicates a statistically significant difference between the two algorithms. The symbols “+”, “=”, and “−” are used to represent that LRMHBA performs better than, equal to, or worse than the comparison algorithms, respectively. The results show that, except for two cases where the *p*-value exceeded 0.05 when compared with LSHADE, all other *p*-values were below 0.05. This indicates that the performance of LRMHBA differs greatly from that of other methods.

### 5.2. Ablation Study

To validate the effectiveness of each improvement strategy in the LRMHBA algorithm, three modified versions of the HBA algorithm were constructed by integrating each of the proposed strategies separately. The descriptions of the three versions are as follows:

- LRMHBA1: HBA combined with Latin hypercube sampling and elite strategy.
- LRMHBA2: HBA combined with a random disturbance strategy.
- LRMHBA3: HBA combined with a staged dual-population co-evolutionary strategy integrating multiple differential evolution variants.

The CEC2017 benchmark suite was used to test HBA, LRMHBA1, LRMHBA2, LRMHBA3, and LRMHBA in the 30-dimensional case. Thirty separate runs of each algorithm were conducted. Table 6 displays the outcomes of the experiment, where the best values among the five algorithms are highlighted in bold. Additionally, the mean ranks and rankings obtained from the Friedman test are provided at the bottom of the table.

From the results, it can be observed that LRMHBA1, LRMHBA2, and LRMHBA3 each achieved the best values for some functions compared to the other algorithms. However, the LRMHBA algorithm, which integrates all three improvement strategies, demonstrated the best overall performance, obtaining the best values for 44 functions and ranking first in the Friedman test’s mean rank.

The experimental results indicate that each strategy contributed to the improvements of the algorithm. Latin hypercube sampling and elite strategy ensured a more uniform population distribution and effectively guided the search process, enhancing search efficiency. The stochastic perturbation strategy prevented premature convergence of the population during the early stages of iteration. Dual-population mutation strategy, which combines multiple differential evolution approaches, adaptively selected different mutation strategies based on the evolutionary process and population fitness. This approach effectively balanced the exploration and exploitation capabilities of the algorithm, thereby improving its global optimization ability.

### 5.3. Exploration and Exploitation Experiment

Exploration refers to the algorithm’s attempt to access new regions in the search space to discover potentially better solutions, while exploitation focuses resources on thoroughly searching a known promising region to find its optimal solution. Excessive exploration may lead to wasted computational resources, whereas excessive exploitation can result in premature convergence to a suboptimal solution, preventing the algorithm from escaping local optima. Consequently, a successful heuristic algorithm needs to properly balance exploration and exploitation.

In this section, a dimensional diversity measurement method [60,61] is employed to better evaluate the ability of LRMHBA to balance exploration and exploitation. The corresponding formula is as follows:

(39) $$Div=\frac{1}{D}\sum_{j=1}^{D}\frac{1}{N}\left(\sum_{i=1}^{N}\left|median\left(x^{j}\right)-x_{i}^{j}\right|\right)$$

(40) $$\;Exploration\%=\frac{Div}{Div_{max}}$$

(41) $$Explotation\%=\frac{\left|Div-{Div}_{max}\right|}{Div_{max}}$$

where Div denotes the diversity value of the population; ${Div}_{max}$ is the maximum diversity value during the iteration. N is the population size; D is the dimensionality of the variables; $x_{i}^{j}$ is the position of the jth dimension of the ith individual; $median\left(x^{j}\right)$ is the median of the jth variable across all individuals in the population.

**Figure 8:**
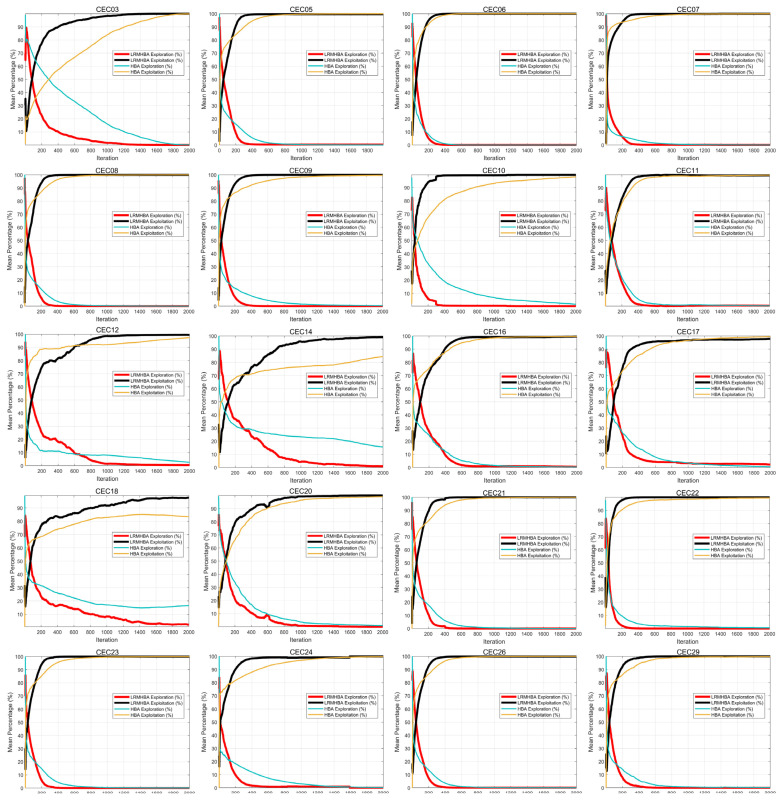
Exploration and exploitation curves of LRMHBA and HBA.

For comparative trials between LRMHBA and HBA, we chose one unimodal function, six simple multimodal functions, seven hybrid functions, and six composition functions from the CEC2017 test suite due to space constraints. The number of iterations was set to 2000, and each algorithm was executed 30 times separately. Figure 8 displays the test findings.

From the Figure 8, it is evident that LRMHBA exhibits strong exploration capability during the early iterations, enabling it to identify potential optimal solutions across various regions of the solution space. As the iterations progress, LRMHBA gradually transitions from exploration to exploitation, refining high-quality solutions and ultimately converging near the global optimum.

Additionally, compared to the HBA algorithm, LRMHBA demonstrates faster convergence, with the population quickly approaching the optimal solution. This indicates that the Latin hypercube sampling method improves population uniformity, enhancing the optimization efficiency of the LRMHBA algorithm. Furthermore, the stochastic perturbation strategy and the dual-population strategy integrating multiple differential mutation methods effectively balance exploration and exploitation, significantly improving the global optimization capability of the algorithm.

## 6. UAV Path Planning Simulation Experiments

In this section, we apply the proposed LRMHBA algorithm to UAV path planning to further analyze its performance.

### 6.1. Experimental Setup

We have constructed three map scenarios of sizes 100 × 100 × 3 km, with increasing complexity. The algorithms selected for comparison, which have shown good performance in this problem, include Particle Swarm Optimization (PSO), Salp swarm algorithm (SSA), Harris hawks optimization (HHO), Dung beetle optimizer (DBO), and Crested Porcupine Optimizer (CPO), as well as the original HBA algorithm and its improved version, SaCHBA_PDN, for 3D UAV path planning. The population size for each algorithm is set to 100, with a maximum of 500 iterations. The parameters for other algorithms are configured according to their respective references, which are shown in Table 7. The map’s starting point is set at (5, 5, 0.3) and the target point at (90, 90, 0.8), with 8 intermediate waypoints. The mountain model and threat area parameters are provided in Table 8. To lessen algorithmic randomness, each algorithm is executed 30 times on its own. The evaluation metrics include the optimal value, average value, variance, and rankings from the Friedman test.

### 6.2. Analysis of Experimental Results

**Figure 9:**
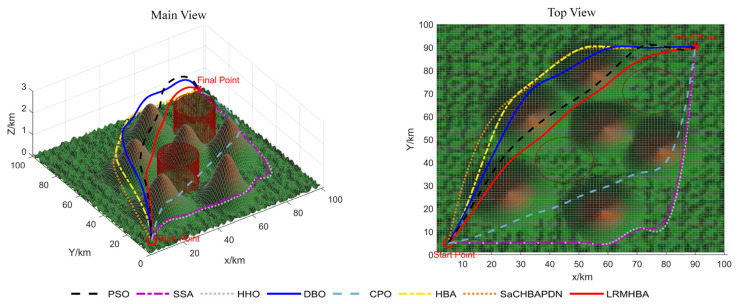
Main and top views of optimal path planning for Scene 1.

**Figure 10:**
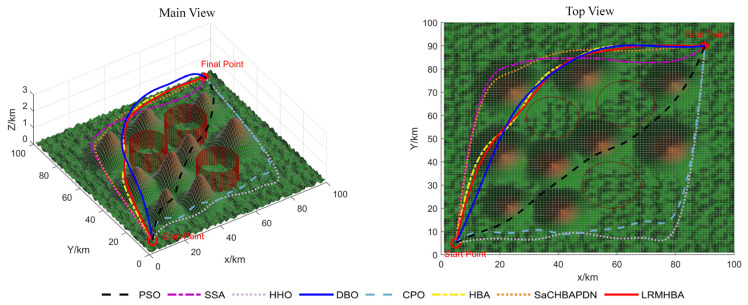
Main and top views of optimal path planning for Scene 2.

**Figure 11:**
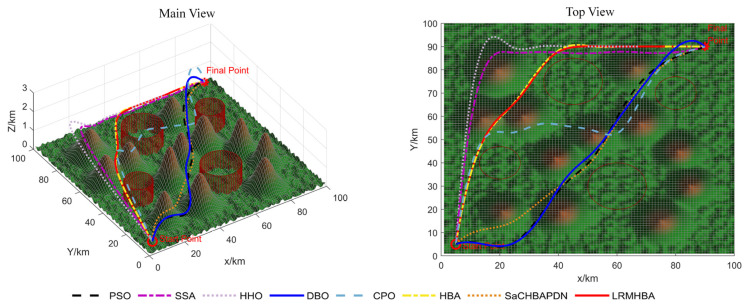
Main and top views of optimal path planning for Scene 3.

**Figure 12:**
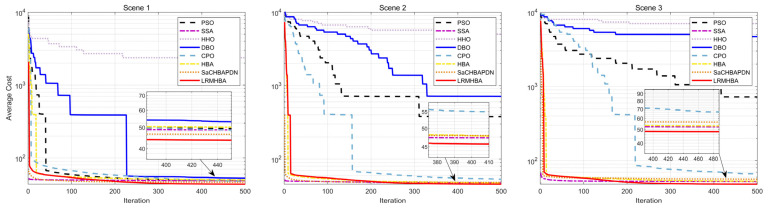
Iterative curves of average flight cost for 3 scenarios.

Figure 9, Figure 10, Figure 11 and Figure 12 display the optimal path planning maps for each of the three scenarios, respectively. Figure 11 is the average cost convergence curve. It is evident that every algorithm was able to identify workable best routes. From Figure 9, we can see that the LRMHBA algorithm found the shortest and smoothest optimal path in the simplest Scenario 1. Although it did not find the shortest path in Scenarios 2 and 3, Figure 10 shows that it achieved the smallest average path planning cost in all three scenarios, indicating that LRMHBA demonstrates excellent stability. Furthermore, in both Scenario 1 and the most complex Scenario 3, LRMHBA outperformed the original HBA algorithm in terms of convergence speed, finding feasible paths with very few iterations. In Scenario 2, while the HBA algorithm showed slightly better convergence speed, it was trapped in a local optimum.

As evidenced in Table 9 summarizing planning outcomes across three scenarios (with bold values indicating optimal metrics), LRMHBA demonstrates superior performance: it achieves the optimal shortest path in Scenario 1, delivers the best average flight cost across all scenarios, and outperforms the original HBA algorithm in most metrics. These findings align with visualizations of optimal trajectory planning and convergence curves. Furthermore, the Sparrow Search Algorithm (SSA) demonstrates the highest stability by consistently identifying feasible paths, while the robustness of HHO, PSO, and DBO has declined. HHO yields excessively high mean values and variances across all scenarios, and both PSO and DBO perform poorly in the complex Scenarios 2 and 3 (with extremely high mean/variance), indicating that these algorithms frequently encounter search failures in challenging environments. Additionally, LRMHBA consistently ranked first in the Friedman test, confirming its excellent adaptability in generating efficient and smooth flight trajectories within both simple and complex three-dimensional obstacle-filled environments.

## 7. Conclusions

This study presents an enhanced Honey Badger Algorithm (LRMHBA) for three-dimensional UAV path planning. The proposed algorithm integrates Latin hypercube sampling with elite preservation mechanisms during population initialization, effectively mitigating low search efficiency resulting from uneven population distribution in conventional random initialization. To strengthen global optimization capacity, a stochastic perturbation mechanism derived from whale optimization is incorporated. Furthermore, an elite-guided dual-population cooperative evolution framework is developed by adaptively combining multiple differential mutation strategies, which dynamically balances global exploration and local exploitation requirements throughout the evolutionary process while ensuring optimization stability.

Comprehensive evaluations on the CEC2017 benchmark suite demonstrate LRMHBA’s superior convergence speed and solution accuracy across low- and high-dimensional optimization tasks, achieving the highest ranking in Friedman’s comprehensive evaluation among comparative powerful algorithms. Statistical validation through Wilcoxon rank-sum tests, ablation analysis, and exploration-exploitation metrics confirms three key aspects: (1) Significant performance differentiation from other algorithms, (2) Demonstrated efficacy of the proposed enhancement strategies, and (3) Better equilibrium between exploration and exploitation capabilities.

Three-dimensional path planning simulations under varying complexity scenarios—encompassing steep terrains and obstacle-threatened airspaces—reveal LRMHBA’s consistent generation of minimal-cost flight trajectories. The algorithm outperforms its counterparts in Friedman rankings, with particular dominance in simplified environments.

Future research directions include dynamic real-time path replanning mechanisms and cooperative multi-UAV optimization frameworks to enhance practical engineering applications. The algorithm’s architectural versatility suggests potential extensions to industrial robotics trajectory optimization, autonomous vehicle navigation, flexible manufacturing scheduling, and computer vision segmentation tasks, demonstrating promising applicability across these domains.

## Figures and Tables

**Table 1:** Latest variants and applications of the HBA algorithm.

Methods	Applications	Authors
Combined quasi-location learning, arbitrarily weighted agents, and adaptive mutation methods.	Selected optimal hyperparameter values for a convolutional neural network CNN applied to sleep apnea diagnosis.	Abasi et al. [25]
Proposed an efficient local search method, called dimensional learning hunting (DLH).	Identified the peak of the global maximum output power of PV cells.	Nassef et al. [26]
Combined HBA with elite backward learning and multidirectional strategies.	Wireless sensor network coverage problem.	Dao et al. [27]
Implemented an Enhanced Solution Quality (ESQ) approach.	Biomedical image segmentation.	Houssei et al. [28]
Developed a new fuzzy deep neural network (FDNN) combined with HBA.	Cloud Computing Privacy Protection Intrusion Detection.	Jain et al. [29]
Proposed a symbiosis-based HBA (SHBA) in conjunction with the cooperative symbio-sis mechanism between honey badgers and honeycreepers.	Engineering problems	Xu et al. [30]
Hybridization of Contrastive Learning with the Honey Badger Algorithm.	Optimization of solar system model parameter values.	Düzenlí et al. [31]
Designed a sparse jNMF method framework guided by the Enhanced Honey Badger Algorithm (EHBA)	Integrated clustering problem	Bansal et al. [32]

**Table 2:** Algorithm parameter settings for CEC2017 benchmark testing.

Algorithms	Parameters	Setting Value
HBA	β (the ability of a honey badger to get food)	6
	C	2
PSO	Cognitive and social factors	$C_{1}=1,C_{2}=1$
DE	Crossover rate	CR=0.5
	Scaling factor	F=0.5
WOA	Fluctuation range	Linear decrease from 2 to 0
AOA	Control parameter	μ=0.499
	Sensitive parameter	α=5
DBO	Disruption factor	k=0.1
	Luminous efficacy	b=0.3
	sensitivity parameter	S=0.5
GQPSO	Inertia weight	Linear decrease from 1 to 0.5
	Cognitive and social factors	$C_{1}=1.5,C_{2}=1.5$
QHDBO	Disruption factor	k=0.1
	Luminous efficacy	b=0.3
	sensitivity parameter	S=0.5
LSHADE	Crossover rate	CR=0.5
	Scaling factor	F=0.5
SaCHBA_PDN	Neighborhood parameter δ	δ=0.01
LRMHBA	Scaling factor	$F_{1}=0.25,F_{2}=\pm 0.5$
	Crossover rate	CR=0.7
	β	6
	C	2

**Table 3:** Test results of LRMHBA with other algorithms on CEC2017 (dim = 30).

Function	Index	HBA	PSO	DE	WOA	AOA	PO	DBO	GQPSO	QHDBO	LSHADE	SaCHBA_PDN	LRMHBA
CEC01	Best	1.338 × 10^+2^	1.450 × 10^+10^	1.524 × 10^+9^	6.486 × 10^+6^	8.346 × 10^+10^	6.412 × 10^+6^	1.057 × 10^+2^	1.853 × 10^+10^	4.951 × 10^+2^	1.017 × 10^+2^	1.039 × 10^+3^	**1.000 × 10^+2^**
	Mean	5.527 × 10^+3^	2.331 × 10^+10^	1.939 × 10^+9^	2.233 × 10^+7^	1.142 × 10^+11^	2.762 × 10^+8^	8.235 × 10^+6^	2.288 × 10^+10^	3.387 × 10^+9^	6.204 × 10^+6^	6.094 × 10^+5^	3.652 × 10^+9^
	Std	5.349 × 10^+3^	4.676 × 10^+9^	1.954 × 10^+8^	1.900 × 10^+7^	1.372 × 10^+10^	3.256 × 10^+8^	1.813 × 10^+7^	1.635 × 10^+9^	3.536 × 10^+9^	2.818 × 10^+7^	1.801 × 10^+6^	2.000 × 10^+10^
	Rank	2	11	9	6	12	7	5	10	8	3	4	1
CEC03	Best	8.876 × 10^+2^	9.232 × 10^+4^	1.096 × 10^+5^	7.498 × 10^+4^	1.849 × 10^+5^	5.820 × 10^+3^	2.776 × 10^+4^	5.180 × 10^+4^	3.618 × 10^+4^	6.933 × 10^+3^	**3.000 × 10^+2^**	**3.000 × 10^+2^**
	Mean	3.116 × 10^+3^	1.384 × 10^+5^	1.687 × 10^+5^	2.196 × 10^+5^	2.536 × 10^+8^	1.470 × 10^+4^	5.913 × 10^+4^	6.176 × 10^+4^	1.809 × 10^+5^	**9.158 × 10^+4^**	3.017 × 10^+2^	7.527 × 10^+2^
	Std	1.773 × 10^+3^	2.807 × 10^+4^	2.485 × 10^+4^	7.259 × 10^+4^	1.199 × 10^+9^	5.493 × 10^+3^	1.606 × 10^+4^	3.754 × 10^+3^	1.103 × 10^+5^	**8.858 × 10^+4^**	3.516 × 10^+0^	8.211 × 10^+2^
	Rank	3	8	10	11	12	4	5	6	9	7	1	2
CEC04	Best	4.600 × 10^+2^	1.171 × 10^+3^	6.183 × 10^+2^	4.808 × 10^+2^	1.267 × 10^+4^	4.849 × 10^+2^	4.769 × 10^+2^	2.829 × 10^+3^	4.968 × 10^+2^	**4.251 × 10^+2^**	4.043 × 10^+2^	4.681 × 10^+2^
	Mean	4.894 × 10^+2^	2.252 × 10^+3^	6.975 × 10^+2^	5.945 × 10^+2^	4.428 × 10^+4^	5.443 × 10^+2^	5.507 × 10^+2^	4.025 × 10^+3^	**9.799 × 10^+2^**	4.286 × 10^+2^	5.154 × 10^+2^	4.923 × 10^+2^
	Std	1.833 × 10^+1^	8.389 × 10^+2^	3.770 × 10^+1^	5.832 × 10^+1^	1.042 × 10^+4^	3.439 × 10^+1^	7.827 × 10^+1^	4.283 × 10^+2^	**5.387 × 10^+2^**	6.277 × 10^+0^	5.107 × 10^+1^	1.530 × 10^+1^
	Rank	2	10	9	7	12	6	5	11	8	1	4	3
CEC05	Best	5.468 × 10^+2^	7.946 × 10^+2^	6.984 × 10^+2^	6.640 × 10^+2^	1.035 × 10^+3^	6.367 × 10^+2^	6.169 × 10^+2^	7.828 × 10^+2^	6.103 × 10^+2^	**5.249 × 10^+2^**	6.135 × 10^+2^	5.298 × 10^+2^
	Mean	6.026 × 10^+2^	8.279 × 10^+2^	7.280 × 10^+2^	7.949 × 10^+2^	1.154 × 10^+3^	7.340 × 10^+2^	7.023 × 10^+2^	8.075 × 10^+2^	6.969 × 10^+2^	6.002 × 10^+2^	6.567 × 10^+2^	**5.646 × 10^+2^**
	Std	2.090 × 10^+1^	1.946 × 10^+1^	**1.121 × 10^+1^**	6.350 × 10^+1^	4.378 × 10^+1^	4.108 × 10^+1^	5.524 × 10^+1^	1.277 × 10^+1^	5.881 × 10^+1^	5.491 × 10^+1^	2.701 × 10^+1^	1.841 × 10^+1^
	Rank	3	11	7	9	12	8	6	10	5	2	4	1
CEC06	Best	6.004 × 10^+2^	6.465 × 10^+2^	6.135 × 10^+2^	6.456 × 10^+2^	7.128 × 10^+2^	6.369 × 10^+2^	6.092 × 10^+2^	6.570 × 10^+2^	6.154 × 10^+2^	**6.000 × 10^+2^**	6.139 × 10^+2^	**6.000 × 10^+2^**
	Mean	6.051 × 10^+2^	6.623 × 10^+2^	6.185 × 10^+2^	6.710 × 10^+2^	7.351 × 10^+2^	6.557 × 10^+2^	6.287 × 10^+2^	6.631 × 10^+2^	6.351 × 10^+2^	**6.002 × 10^+2^**	6.315 × 10^+2^	6.042 × 10^+2^
	Std	4.823 × 10^+0^	7.407 × 10^+0^	1.804 × 10^+0^	1.295 × 10^+1^	9.505 × 10^+0^	8.484 × 10^+0^	8.472 × 10^+0^	2.505 × 10^+0^	2.201 × 10^+1^	**7.372 × 10^−1^**	7.130 × 100	2.317 × 10^+1^
	Rank	3	9	4	11	12	8	5	10	6	1	7	2
CEC07	Best	8.016 × 10^+2^	1.507 × 10^+3^	1.011 × 10^+3^	1.022 × 10^+3^	2.891 × 10^+3^	9.921 × 10^+2^	8.211 × 10^+2^	1.130 × 10^+3^	7.996 × 10^+2^	7.614 × 10^+2^	8.541 × 10^+2^	**7.569 × 10^+2^**
	Mean	8.637 × 10^+2^	1.781 × 10^+3^	1.084 × 10^+3^	1.230 × 10^+3^	3.254 × 10^+3^	1.146 × 10^+3^	9.310 × 10^+2^	1.155 × 10^+3^	8.807 × 10^+2^	8.447 × 10^+2^	9.773 × 10^+2^	**7.849 × 10^+2^**
	Std	4.823 × 10^+0^	7.407 × 10^+0^	1.804 × 10^+0^	1.295 × 10^+1^	9.505 × 10^+0^	8.484 × 10^+0^	8.472 × 10^+0^	2.505 × 10^+0^	2.201 × 10^+1^	**7.372 × 10^−1^**	7.130 × 10^+0^	2.317 × 10^+1^
	Rank	3	11	7	10	12	8	5	9	4	2	6	1
CEC08	Best	8.497 × 10^+2^	1.097 × 10^+3^	1.003 × 10^+3^	9.079 × 10^+2^	1.301 × 10^+3^	9.283 × 10^+2^	9.194 × 10^+2^	1.037 × 10^+3^	8.948 × 10^+2^	8.289 × 10^+2^	8.766 × 10^+2^	**8.259 × 10^+2^**
	Mean	8.952 × 10^+2^	1.137 × 10^+3^	1.036 × 10^+3^	1.009 × 10^+3^	1.384 × 10^+3^	9.754 × 10^+2^	1.003 × 10^+3^	1.056 × 10^+3^	9.682 × 10^+2^	8.931 × 10^+2^	9.267 × 10^+2^	**8.641 × 10^+2^**
	Std	1.728 × 10^+1^	2.386 × 10^+1^	1.398 × 10^+1^	5.327 × 10^+1^	4.284 × 10^+1^	2.950 × 10^+1^	4.809 × 10^+1^	**1.097 × 10^+1^**	4.448 × 10^+1^	6.405 × 10^+1^	2.958 × 10^+1^	2.101 × 10^+1^
	Rank	2	11	9	7	12	6	8	10	5	3	4	1
CEC09	Best	1.321 × 10^+3^	7.533 × 10^+3^	6.338 × 10^+3^	5.233 × 10^+3^	1.936 × 10^+4^	2.744 × 10^+3^	1.501 × 10^+3^	5.443 × 10^+3^	1.680 × 10^+3^	**9.000 × 10^+2^**	1.713 × 10^+3^	9.001 × 10^+2^
	Mean	1.926 × 10^+3^	1.121 × 10^+4^	8.499 × 10^+3^	8.871 × 10^+3^	3.443 × 10^+4^	5.213 × 10^+3^	5.083 × 10^+3^	6.333 × 10^+3^	4.394 × 10^+3^	9.826 × 10^+2^	3.040 × 10^+3^	**9.135 × 10^+2^**
	Std	5.932 × 10^+2^	2.369 × 10^+3^	9.801 × 10^+2^	3.239 × 10^+3^	5.488 × 10^+3^	1.076 × 10^+3^	2.136 × 10^+3^	4.551 × 10^+2^	1.989 × 10^+3^	2.910 × 10^+2^	9.291 × 10^+2^	**3.453 × 10^+1^**
	Rank	3	11	10	9	12	7	6	8	5	2	4	1
CEC10	Best	**3.510 × 10^+3^**	7.510 × 10^+3^	5.703 × 10^+3^	5.186 × 10^+3^	9.690 × 10^+3^	3.960 × 10^+3^	4.037 × 10^+3^	7.336 × 10^+3^	5.674 × 10^+3^	4.053 × 10^+3^	4.315 × 10^+3^	3.634 × 10^+3^
	Mean	4.973 × 10^+3^	8.093 × 10^+3^	6.232 × 10^+3^	6.530 × 10^+3^	1.060 × 10^+4^	5.771 × 10^+3^	5.190 × 10^+3^	7.948 × 10^+3^	6.834 × 10^+3^	5.701 × 10^+3^	5.459 × 10^+3^	**4.944 × 10^+3^**
	Std	1.028 × 10^+3^	3.292 × 10^+2^	2.571 × 10^+2^	8.146 × 10^+2^	4.135 × 10^+2^	7.682 × 10^+2^	5.156 × 10^+2^	**2.495 × 10^+2^**	6.093 × 10^+2^	1.084 × 10^+3^	7.670 × 10^+2^	7.891 × 10^+2^
	Rank	2	11	7	8	12	6	3	10	9	5	4	1
CEC11	Best	1.135 × 10^+3^	2.200 × 10^+3^	1.933 × 10^+3^	1.405 × 10^+3^	1.315 × 10^+4^	1.247 × 10^+3^	1.252 × 10^+3^	2.618 × 10^+3^	1.297 × 10^+3^	1.119 × 10^+3^	1.185 × 10^+3^	**1.113 × 10^+3^**
	Mean	1.221 × 10^+3^	5.106 × 10^+3^	3.990 × 10^+3^	1.823 × 10^+3^	4.718 × 10^+4^	1.387 × 10^+3^	1.494 × 10^+3^	3.296 × 10^+3^	3.020 × 10^+3^	1.275 × 10^+3^	1.281 × 10^+3^	**1.146 × 10^+3^**
	Std	5.590 × 10^+1^	2.024 × 10^+3^	1.279 × 10^+3^	4.804 × 10^+2^	2.858 × 10^+4^	8.294 × 10^+1^	1.176 × 10^+2^	2.900 × 10^+2^	5.266 × 10^+3^	6.030 × 10^+2^	5.972 × 10^+1^	**2.992 × 10^+1^**
	Rank	3	11	10	8	12	5	6	9	7	2	4	1
CEC12	Best	1.592 × 10^+4^	7.208 × 10^+8^	4.120 × 10^+7^	1.760 × 10^+7^	1.363 × 10^+10^	4.470 × 10^+6^	4.568 × 10^+5^	3.117 × 10^+9^	9.469 × 10^+4^	1.103 × 10^+5^	2.885 × 10^+4^	**4.490 × 10^+3^**
	Mean	7.742 × 10^+4^	1.373 × 10^+9^	8.865 × 10^+7^	1.647 × 10^+8^	2.275 × 10^+10^	6.751 × 10^+7^	2.423 × 10^+7^	4.126 × 10^+9^	5.908 × 10^+8^	7.797 × 10^+6^	8.686 × 10^+5^	**2.879 × 10^+4^**
	Std	5.058 × 10^+4^	3.633 × 10^+8^	2.276 × 10^+7^	1.480 × 10^+8^	5.438 × 10^+9^	6.930 × 10^+7^	5.387 × 10^+7^	5.231 × 10^+8^	8.997 × 10^+8^	1.550 × 10^+7^	1.194 × 10^+6^	**1.827 × 10^+4^**
	Rank	2	10	8	9	12	6	5	11	7	4	3	1
CEC13	Best	3.514 × 10^+3^	1.251 × 10^+8^	4.526 × 10^+6^	5.675 × 10^+4^	8.252 × 10^+9^	8.910 × 10^+3^	1.929 × 10^+4^	8.200 × 10^+8^	9.344 × 10^+4^	2.978 × 10^+3^	1.135 × 10^+4^	**1.332 × 10^+3^**
	Mean	**3.605 × 10^+4^**	4.400 × 10^+8^	1.906 × 10^+7^	5.387 × 10^+5^	2.346 × 10^+10^	1.099 × 10^+5^	2.589 × 10^+6^	1.758 × 10^+9^	3.936 × 10^+7^	1.649 × 10^+5^	2.576 × 10^+5^	6.184 × 10^+8^
	Std	**2.727 × 10^+4^**	2.874 × 10^+8^	8.011 × 10^+6^	9.394 × 10^+5^	8.292 × 10^+9^	7.376 × 10^+4^	8.975 × 10^+6^	4.847 × 10^+8^	1.926 × 10^+8^	3.787 × 10^+5^	8.169 × 10^+5^	3.387 × 10^+9^
	Rank	2	10	9	7	12	5	6	11	8	3	4	1
CEC14	Best	1.827 × 10^+3^	5.271 × 10^+4^	7.695 × 10^+4^	4.459 × 10^+3^	5.772 × 10^+6^	4.454 × 10^+3^	2.475 × 10^+3^	1.644 × 10^+5^	2.050 × 10^+3^	**1.430 × 10^+3^**	1.627 × 10^+3^	1.529 × 10^+3^
	Mean	7.510 × 10^+3^	3.906 × 10^+5^	4.580 × 10^+5^	1.956 × 10^+6^	4.274 × 10^+7^	6.359 × 10^+4^	1.120 × 10^+5^	9.828 × 10^+5^	6.722 × 10^+6^	2.346 × 10^+4^	**1.747 × 10^+3^**	3.260 × 10^+3^
	Std	8.299 × 10^+3^	2.459 × 10^+5^	2.379 × 10^+5^	1.731 × 10^+6^	2.947 × 10^+7^	4.375 × 10^+4^	2.986 × 10^+5^	3.588 × 10^+5^	1.762 × 10^+7^	6.919 × 10^+4^	**1.104 × 10^+2^**	2.250 × 10^+3^
	Rank	4	8	9	10	12	6	5	11	7	2	1	3
CEC15	Best	1.756 × 10^+3^	1.216 × 10^+7^	1.870 × 10^+5^	2.030 × 10^+4^	1.854 × 10^+9^	1.512 × 10^+4^	5.238 × 10^+3^	2.545 × 10^+6^	4.536 × 10^+3^	1.656 × 10^+3^	2.630 × 10^+3^	**1.552 × 10^+3^**
	Mean	1.509 × 10^+4^	8.185 × 10^+7^	2.507 × 10^+6^	1.764 × 10^+5^	5.448 × 10^+9^	7.030 × 10^+4^	7.602 × 10^+4^	1.161 × 10^+7^	3.015 × 10^+7^	8.211 × 10^+4^	**1.387 × 10^+4^**	1.083 × 10^+8^
	Std	1.401 × 10^+4^	5.172 × 10^+7^	1.403 × 10^+6^	2.323 × 10^+5^	2.303 × 10^+9^	5.388 × 10^+4^	8.097 × 10^+4^	6.087 × 10^+6^	1.648 × 10^+8^	2.095 × 10^+5^	**1.248 × 10^+4^**	5.933 × 10^+8^
	Rank	2	11	9	8	12	7	6	10	5	4	3	1
CEC16	Best	1.967 × 10^+3^	2.894 × 10^+3^	2.758 × 10^+3^	2.985 × 10^+3^	6.039 × 10^+3^	2.541 × 10^+3^	2.397 × 10^+3^	3.699 × 10^+3^	2.549 × 10^+3^	2.116 × 10^+3^	2.107 × 10^+3^	**1.745 × 10^+3^**
	Mean	2.545 × 10^+3^	3.705 × 10^+3^	3.015 × 10^+3^	3.753 × 10^+3^	8.254 × 10^+3^	3.200 × 10^+3^	2.992 × 10^+3^	4.070 × 10^+3^	3.478 × 10^+3^	2.782 × 10^+3^	2.749 × 10^+3^	**2.394 × 10^+3^**
	Std	2.814 × 10^+2^	3.663 × 10^+2^	**1.557 × 10^+2^**	4.069 × 10^+2^	1.447 × 10^+3^	3.791 × 10^+2^	3.697 × 10^+2^	1.822 × 10^+2^	5.077 × 10^+2^	4.859 × 10^+2^	3.384 × 10^+2^	2.963 × 10^+2^
	Rank	2	10	6	9	12	7	5	11	8	4	3	1
CEC17	Best	1.773 × 10^+3^	2.445 × 10^+3^	2.006 × 10^+3^	1.859 × 10^+3^	4.420 × 10^+3^	2.024 × 10^+3^	1.917 × 10^+3^	2.396 × 10^+3^	2.379 × 10^+3^	1.775 × 10^+3^	2.011 × 10^+3^	**1.748 × 10^+3^**
	Mean	2.111 × 10^+3^	2.800 × 10^+3^	2.256 × 10^+3^	2.615 × 10^+3^	1.542 × 10^+4^	2.468 × 10^+3^	2.390 × 10^+3^	2.716 × 10^+3^	4.346 × 10^+3^	2.021 × 10^+3^	2.360 × 10^+3^	**1.992 × 10^+3^**
	Std	2.067 × 10^+2^	1.893 × 10^+2^	**1.206 × 10^+2^**	3.292 × 10^+2^	1.574 × 10^+4^	2.095 × 10^+2^	2.477 × 10^+2^	1.344 × 10^+2^	4.889 × 10^+3^	1.896 × 10^+2^	2.277 × 10^+2^	1.365 × 10^+2^
	Rank	3	10	4	8	12	7	6	9	11	2	5	1
CEC18	Best	3.781 × 10^+4^	1.043 × 10^+6^	6.850 × 10^+5^	1.258 × 10^+5^	1.072 × 10^+8^	5.909 × 10^+4^	5.143 × 10^+4^	2.027 × 10^+6^	4.925 × 10^+4^	**2.729 × 10^+3^**	1.453 × 10^+4^	1.169 × 10^+4^
	Mean	1.607 × 10^+5^	7.478 × 10^+6^	2.193 × 10^+6^	4.392 × 10^+6^	5.464 × 10^+8^	9.443 × 10^+5^	1.848 × 10^+6^	5.085 × 10^+6^	2.416 × 10^+7^	6.476 × 10^+5^	**4.699 × 10^+4^**	1.085 × 10^+7^
	Std	9.438 × 10^+4^	4.572 × 10^+6^	7.608 × 10^+5^	5.557 × 10^+6^	3.452 × 10^+8^	7.713 × 10^+5^	4.402 × 10^+6^	1.571 × 10^+6^	7.360 × 10^+7^	6.789 × 10^+5^	**2.867 × 10^+4^**	5.891 × 10^+7^
	Rank	3	11	9	8	12	7	4	10	6	5	1	2
CEC19	Best	2.027 × 10^+3^	2.395 × 10^+7^	4.237 × 10^+5^	3.315 × 10^+5^	1.356 × 10^+9^	4.861 × 10^+3^	2.219 × 10^+3^	3.061 × 10^+7^	3.177 × 10^+3^	1.912 × 10^+3^	2.092 × 10^+3^	**1.916 × 10^+3^**
	Mean	**9.046 × 10^+3^**	1.531 × 10^+8^	1.886 × 10^+6^	4.444 × 10^+6^	6.274 × 10^+9^	1.143 × 10^+6^	3.107 × 10^+6^	5.912 × 10^+7^	4.851 × 10^+7^	9.768 × 10^+3^	1.695 × 10^+4^	9.710 × 10^+3^
	Std	**1.143 × 10^+4^**	6.952 × 10^+7^	1.010 × 10^+6^	4.071 × 10^+6^	2.884 × 10^+9^	8.138 × 10^+5^	1.492 × 10^+7^	1.993 × 10^+7^	7.002 × 10^+7^	1.688 × 10^+4^	1.607 × 10^+4^	1.209 × 10^+4^
	Rank	2	11	7	9	12	6	5	10	8	1	4	3
CEC20	Best	2.166 × 10^+3^	2.512 × 10^+3^	2.229 × 10^+3^	2.268 × 10^+3^	3.293 × 10^+3^	2.268 × 10^+3^	2.315 × 10^+3^	2.486 × 10^+3^	2.209 × 10^+3^	2.040 × 10^+3^	2.385 × 10^+3^	**2.034 × 10^+3^**
	Mean	2.406 × 10^+3^	2.781 × 10^+3^	2.484 × 10^+3^	2.708 × 10^+3^	3.737 × 10^+3^	2.561 × 10^+3^	2.683 × 10^+3^	2.610 × 10^+3^	3.171 × 10^+3^	**2.278 × 10^+3^**	2.661 × 10^+3^	2.425 × 10^+3^
	Std	1.944 × 10^+2^	1.354 × 10^+2^	1.198 × 10^+2^	2.352 × 10^+2^	2.031 × 10^+2^	1.666 × 10^+2^	1.890 × 10^+2^	**5.726 × 10^+1^**	4.238 × 10^+2^	2.131 × 10^+2^	1.726 × 10^+2^	2.705 × 10^+2^
	Rank	2	10	4	8	12	5	9	6	11	1	7	3
CEC21	Best	2.345 × 10^+3^	2.571 × 10^+3^	2.462 × 10^+3^	2.485 × 10^+3^	2.798 × 10^+3^	2.414 × 10^+3^	2.415 × 10^+3^	2.546 × 10^+3^	2.481 × 10^+3^	**2.328 × 10^+3^**	2.393 × 10^+3^	2.331 × 10^+3^
	Mean	2.382 × 10^+3^	2.612 × 10^+3^	2.520 × 10^+3^	2.593 × 10^+3^	2.907 × 10^+3^	2.506 × 10^+3^	2.495 × 10^+3^	2.586 × 10^+3^	2.663 × 10^+3^	2.411 × 10^+3^	2.452 × 10^+3^	**2.376 × 10^+3^**
	Std	2.473 × 10^+1^	2.137 × 10^+1^	1.868 × 10^+1^	5.435 × 10^+1^	5.768 × 10^+1^	4.985 × 10^+1^	3.936 × 10^+1^	**1.499 × 10^+1^**	9.272 × 10^+1^	6.729 × 10^+1^	3.647 × 10^+1^	1.127 × 10^+2^
	Rank	2	10	7	9	12	6	5	8	11	3	4	1
CEC22	Best	2.300 × 10^+3^	4.134 × 10^+3^	3.936 × 10^+3^	2.418 × 10^+3^	9.698 × 10^+3^	2.355 × 10^+3^	2.308 × 10^+3^	4.322 × 10^+3^	1.404 × 10^+4^	2.300 × 10^+3^	2.300 × 10^+3^	**2.300 × 10^+3^**
	Mean	4.142 × 10^+3^	7.331 × 10^+3^	5.891 × 10^+3^	8.334 × 10^+3^	1.178 × 10^+4^	**3.701 × 10^+3^**	5.025 × 10^+3^	4.801 × 10^+3^	1.404 × 10^+4^	6.053 × 10^+3^	4.028 × 10^+3^	4.236 × 10^+3^
	Std	2.581 × 10^+3^	2.264 × 10^+3^	1.156 × 10^+3^	1.582 × 10^+3^	6.589 × 10^+2^	1.963 × 10^+3^	2.079 × 10^+3^	1.910 × 10^+2^	**7.400 × 10^−12^**	2.830 × 10^+3^	2.313 × 10^+3^	2.206 × 10^+3^
	Rank	2	9	7	10	11	4	6	5	12	8	3	1
CEC23	Best	2.700 × 10^+3^	2.855 × 10^+3^	2.795 × 10^+3^	2.961 × 10^+3^	3.320 × 10^+3^	2.802 × 10^+3^	2.761 × 10^+3^	3.141 × 10^+3^	2.969 × 10^+3^	2.672 × 10^+3^	2.752 × 10^+3^	**2.661 × 10^+3^**
	Mean	2.755 × 10^+3^	3.019 × 10^+3^	2.843 × 10^+3^	3.144 × 10^+3^	3.854 × 10^+3^	2.987 × 10^+3^	2.869 × 10^+3^	3.181 × 10^+3^	3.348 × 10^+3^	2.737 × 10^+3^	2.889 × 10^+3^	**2.706 × 10^+3^**
	Std	2.428 × 10^+1^	7.029 × 10^+1^	**1.291 × 10^+1^**	1.059 × 10^+2^	2.065 × 10^+2^	8.730 × 10^+1^	5.319 × 10^+1^	1.959 × 10^+1^	2.544 × 10^+2^	5.876 × 10^+1^	8.346 × 10^+1^	1.829 × 10^+1^
	Rank	3	8	4	9	12	7	5	10	11	2	6	1
CEC24	Best	2.867 × 10^+3^	3.059 × 10^+3^	3.028 × 10^+3^	3.112 × 10^+3^	3.719 × 10^+3^	2.999 × 10^+3^	2.937 × 10^+3^	3.315 × 10^+3^	4.689 × 10^+3^	2.853 × 10^+3^	2.945 × 10^+3^	**2.854 × 10^+3^**
	Mean	2.945 × 10^+3^	3.127 × 10^+3^	3.055 × 10^+3^	3.300 × 10^+3^	4.223 × 10^+3^	3.114 × 10^+3^	3.031 × 10^+3^	3.427 × 10^+3^	4.689 × 10^+3^	2.943 × 10^+3^	3.098 × 10^+3^	**2.884 × 10^+3^**
	Std	6.015 × 10^+1^	4.252 × 10^+1^	1.365 × 10^+1^	1.278 × 10^+2^	2.508 × 10^+2^	7.195 × 10^+1^	5.458 × 10^+1^	3.559 × 10^+1^	**9.250 × 10^−13^**	5.920 × 10^+1^	1.074 × 10^+2^	2.135 × 10^+1^
	Rank	3	8	5	9	11	7	4	10	12	2	6	1
CEC25	Best	2.884 × 10^+3^	3.955 × 10^+3^	3.068 × 10^+3^	2.895 × 10^+3^	1.190 × 10^+4^	2.903 × 10^+3^	2.888 × 10^+3^	3.221 × 10^+3^	2.884 × 10^+3^	2.878 × 10^+3^	2.884 × 10^+3^	**2.883 × 10^+3^**
	Mean	2.891 × 10^+3^	4.516 × 10^+3^	3.184 × 10^+3^	2.968 × 10^+3^	1.622 × 10^+4^	2.964 × 10^+3^	2.941 × 10^+3^	3.321 × 10^+3^	2.968 × 10^+3^	**2.880 × 10^+3^**	2.909 × 10^+3^	3.270 × 10^+3^
	Std	1.270 × 10^+1^	4.659 × 10^+2^	5.414 × 10^+1^	3.602 × 10^+1^	2.442 × 10^+3^	3.968 × 10^+1^	4.287 × 10^+1^	3.945 × 10^+1^	9.264 × 10^+1^	**3.626 × 10^+0^**	2.385 × 10^+1^	2.096 × 10^+3^
	Rank	3	11	9	7	12	8	6	10	5	1	4	2
CEC26	Best	**2.800 × 10^+3^**	6.034 × 10^+3^	5.406 × 10^+3^	3.570 × 10^+3^	1.238 × 10^+4^	3.267 × 10^+3^	3.147 × 10^+3^	6.451 × 10^+3^	2.412 × 10^+4^	3.490 × 10^+3^	4.933 × 10^+3^	2.900 × 10^+3^
	Mean	4.670 × 10^+3^	7.175 × 10^+3^	5.730 × 10^+3^	7.754 × 10^+3^	1.526 × 10^+4^	6.501 × 10^+3^	6.149 × 10^+3^	7.665 × 10^+3^	2.412 × 10^+4^	**4.254 × 10^+3^**	5.691 × 10^+3^	4.577 × 10^+3^
	Std	7.419 × 10^+2^	5.713 × 10^+2^	1.491 × 10^+2^	1.588 × 10^+3^	1.535 × 10^+3^	1.605 × 10^+3^	8.762 × 10^+2^	6.018 × 10^+2^	**1.110 × 10^−11^**	6.852 × 10^+2^	5.225 × 10^+2^	2.241 × 10^+3^
	Rank	3	8	5	9	11	7	6	10	12	2	4	1
CEC27	Best	**3.186 × 10^+3^**	3.234 × 10^+3^	3.221 × 10^+3^	3.200 × 10^+3^	4.455 × 10^+3^	3.243 × 10^+3^	3.210 × 10^+3^	3.563 × 10^+3^	3.220 × 10^+3^	3.200 × 10^+3^	3.224 × 10^+3^	3.201 × 10^+3^
	Mean	3.289 × 10^+3^	3.327 × 10^+3^	3.231 × 10^+3^	3.200 × 10^+3^	5.304 × 10^+3^	3.326 × 10^+3^	3.257 × 10^+3^	3.637 × 10^+3^	3.385 × 10^+3^	**3.200 × 10^+3^**	3.313 × 10^+3^	3.343 × 10^+3^
	Std	7.288 × 10^+1^	5.794 × 10^+1^	4.675 × 10^+0^	**1.870 × 10^−4^**	5.052 × 10^+2^	6.417 × 10^+1^	3.763 × 10^+1^	3.376 × 10^+1^	1.542 × 10^+2^	2.595 × 10^−4^	6.824 × 10^+1^	4.433 × 10^+2^
	Rank	6	9	3	2	12	8	5	11	10	1	7	4
CEC28	Best	3.108 × 10^+3^	3.899 × 10^+3^	3.477 × 10^+3^	3.296 × 10^+3^	8.198 × 10^+3^	3.292 × 10^+3^	3.259 × 10^+3^	4.467 × 10^+3^	3.230 × 10^+3^	3.300 × 10^+3^	3.192 × 10^+3^	3.163 × 10^+3^
	Mean	3.215 × 10^+3^	4.752 × 10^+3^	3.707 × 10^+3^	3.299 × 10^+3^	1.196 × 10^+4^	3.363 × 10^+3^	3.365 × 10^+3^	4.684 × 10^+3^	4.067 × 10^+3^	3.300 × 10^+3^	3.219 × 10^+3^	3.210 × 10^+3^
	Std	3.204 × 10^+1^	6.589 × 10^+2^	1.144 × 10^+2^	1.109 × 10^+0^	1.963 × 10^+3^	3.915 × 10^+1^	1.320 × 10^+2^	8.757 × 10^+1^	8.724 × 10^+2^	2.982 × 10^−4^	2.089 × 10^+1^	2.617 × 10^+1^
	Rank	2	10	8	5	12	7	6	11	9	4	3	1
CEC29	Best	3.383 × 10^+03^	4.081 × 10^+3^	3.669 × 10^+3^	3.910 × 10^+3^	7.304 × 10^+3^	4.149 × 10^+3^	3.544 × 10^+3^	4.324 × 10^+3^	4.168 × 10^+3^	**3.145 × 10^+3^**	3.618 × 10^+3^	3.291 × 10^+3^
	Mean	4.029 × 10^+03^	4.597 × 10^+3^	3.978 × 10^+3^	4.686 × 10^+3^	2.117 × 10^+4^	4.606 × 10^+3^	4.106 × 10^+3^	4.803 × 10^+3^	5.665 × 10^+3^	**3.613 × 10^+3^**	4.242 × 10^+3^	3.664 × 10^+3^
	Std	3.881 × 10^+02^	3.115 × 10^+2^	**1.391 × 10^+2^**	4.986 × 10^+2^	1.387 × 10^+4^	2.055 × 10^+2^	2.676 × 10^+2^	1.642 × 10^+2^	2.420 × 10^+3^	2.993 × 10^+2^	2.649 × 10^+2^	1.863 × 10^+2^
	Rank	4	9	3	7	12	8	5	10	11	1	6	2
CEC30	Best	6.126 × 10^+3^	2.321 × 10^+7^	2.587 × 10^+5^	9.263 × 10^+3^	4.416 × 10^+8^	5.747 × 10^+5^	9.088 × 10^+3^	1.522 × 10^+8^	1.185 × 10^+4^	**3.212 × 10^+3^**	8.460 × 10^+3^	5.386 × 10^+3^
	Mean	5.553 × 10^+5^	6.233 × 10^+7^	1.013 × 10^+6^	1.001 × 10^+7^	3.003 × 10^+9^	9.539 × 10^+6^	1.222 × 10^+6^	2.894 × 10^+8^	5.547 × 10^+5^	1.396 × 10^+4^	7.144 × 10^+4^	**8.781 × 10^+3^**
	Std	2.808 × 10^+6^	2.660 × 10^+7^	4.585 × 10^+5^	1.097 × 10^+7^	1.539 × 10^+9^	6.865 × 10^+6^	2.038 × 10^+6^	6.587 × 10^+7^	7.628 × 10^+5^	2.534 × 10^+4^	7.037 × 10^+4^	**3.112 × 10^+3^**
	Rank	3	10	7	8	12	9	6	11	5	1	4	2
Mean Rank		2.72	9.90	7.07	8.17	11.90	6.62	5.48	9.59	8.10	2.72	4.14	1.58
Final Ranking		2	11	7	9	12	6	5	10	8	2	4	1

**Table 4:** Test results of LRMHBA with other algorithms on CEC2017 (dim = 100).

Function	Index	HBA	PSO	DE	WOA	AOA	PO	DBO	GQPSO	QHDBO	LSHADE	SaCHBA_PDN	LRMHBA
CEC01	Best	1.822 × 10^+7^	2.767 × 10^+11^	1.773 × 10^+11^	2.415 × 10^+9^	5.206 × 10^+11^	2.056 × 10^+10^	3.831 × 10^+9^	1.623 × 10^+11^	7.078 × 10^+09^	**5.225 × 10^+3^**	2.535 × 10^+7^	1.166 × 10^+5^
	Mean	1.558 × 10^+9^	3.326 × 10^+11^	2.199 × 10^+11^	3.875 × 10^+9^	6.059 × 10^+11^	3.809 × 10^+10^	1.673 × 10^+10^	1.657 × 10^+11^	3.036 × 10^+10^	2.716 × 10^+9^	3.066 × 10^+8^	**1.247 × 10^+6^**
	Std	1.853 × 10^+9^	3.193 × 10^+10^	1.241 × 10^+10^	1.026 × 10^+9^	3.294 × 10^+10^	9.365 × 10^+9^	2.158 × 10^+10^	1.802 × 10^+9^	1.202 × 10^+10^	8.337 × 10^+9^	5.054 × 10^+8^	**1.138 × 10^+6^**
	Rank	4	11	10	5	12	8	6	9	7	2	3	1
CEC03	Best	2.197 × 10^+5^	7.104 × 10^+5^	7.156 × 10^+5^	4.868 × 10^+5^	8.023 × 10^+5^	1.715 × 10^+5^	3.466 × 10^+5^	2.649 × 10^+5^	3.761 × 10^+5^	2.962 × 10^+5^	**6.301 × 10^+4^**	1.655 × 10^+5^
	Mean	2.722 × 10^+5^	9.684 × 10^+5^	8.882 × 10^+5^	8.701 × 10^+5^	8.249 × 10^+10^	2.044 × 10^+5^	6.375 × 10^+5^	2.872 × 10^+5^	1.184 × 10^+6^	8.650 × 10^+5^	**8.665 × 10^+4^**	2.059 × 10^+5^
	Std	2.239 × 10^+4^	1.329 × 10^+5^	6.350 × 10^+4^	1.978 × 10^+5^	2.661 × 10^+11^	1.588 × 10^+4^	2.324 × 10^+5^	**9.710 × 10^+3^**	3.119 × 10^+5^	3.466 × 10^+5^	1.451 × 10^+4^	2.187 × 10^+4^
	Rank	4	10	8	7	12	2	6	5	11	9	1	3
CEC04	Best	7.874 × 10^+2^	3.907 × 10^+4^	3.310 × 10^+4^	1.534 × 10^+03^	1.894 × 10^+5^	2.208 × 10^+3^	1.281 × 10^+3^	3.136 × 10^+4^	2.113 × 10^+3^	**4.976 × 10^+2^**	7.942 × 10^+2^	7.001 × 10^+2^
	Mean	9.253 × 10^+2^	6.342 × 10^+4^	3.802 × 10^+4^	2.410 × 10^+03^	2.693 × 10^+5^	3.864 × 10^+3^	3.021 × 10^+3^	3.438 × 10^+4^	6.928 × 10^+3^	2.372 × 10^+3^	9.884 × 10^+2^	**7.725 × 10^+2^**
	Std	9.496 × 10^+1^	1.704 × 10^+4^	2.411 × 10^+3^	5.914 × 10^+02^	3.821 × 10^+4^	8.715 × 10^+2^	2.804 × 10^+3^	1.233 × 10^+3^	4.368 × 10^+3^	8.155 × 10^+3^	1.547 × 10^+2^	**4.891 × 10^+1^**
	Rank	3	11	10	5	12	7	6	9	8	2	4	1
CEC05	Best	9.526 × 10^+2^	2.046 × 10^+3^	2.101 × 10^+3^	1.443 × 10^+03^	2.830 × 10^+3^	1.459 × 10^+3^	1.249 × 10^+3^	1.801 × 10^+3^	1.180 × 10^+3^	**6.224 × 10^+2^**	1.147 × 10^+3^	7.390 × 10^+2^
	Mean	1.109 × 10^+3^	2.345 × 10^+3^	2.163 × 10^+3^	1.662 × 10^+03^	3.027 × 10^+3^	1.554 × 10^+3^	1.666 × 10^+3^	1.835 × 10^+3^	1.328 × 10^+3^	1.243 × 10^+3^	1.288 × 10^+3^	**8.854 × 10^+2^**
	Std	8.968 × 10^+1^	1.271 × 10^+2^	3.802 × 10^+1^	1.815 × 10^+02^	1.081 × 10^+2^	6.241 × 10^+1^	1.425 × 10^+2^	**1.675 × 10^+1^**	6.349 × 10^+1^	3.053 × 10^+2^	7.608 × 10^+1^	6.979 × 10^+1^
	Rank	2	11	10	7	12	6	8	9	5	3	4	1
CEC06	Best	6.258 × 10^+2^	7.132 × 10^+2^	6.775 × 10^+2^	6.784 × 10^+02^	7.473 × 10^+2^	6.719 × 10^+2^	6.477 × 10^+2^	6.856 × 10^+2^	6.481 × 10^+2^	**6.000 × 10^+2^**	6.549 × 10^+2^	6.010 × 10^+2^
	Mean	6.375 × 10^+2^	7.247 × 10^+2^	6.858 × 10^+2^	6.927 × 10^+02^	7.609 × 10^+2^	6.812 × 10^+2^	6.727 × 10^+2^	6.906 × 10^+2^	6.546 × 10^+2^	6.149 × 10^+2^	6.634 × 10^+2^	**6.031 × 10^+2^**
	Std	7.000 × 10^+0^	6.883 × 10^+0^	3.096 × 10^+0^	9.670 × 10^+00^	6.521 × 10^+0^	5.067 × 10^+0^	9.215 × 10^+0^	2.162 × 10^+0^	4.094 × 10^+0^	2.964 × 10^+1^	4.470 × 10^+0^	**1.402 × 10^+0^**
	Rank	3	11	8	9	12	7	6	10	4	2	5	1
CEC07	Best	1.637 × 10^+3^	6.805 × 10^+3^	8.288 × 10^+3^	3.130 × 10^+3^	1.200 × 10^+4^	2.964 × 10^+3^	1.844 × 10^+3^	3.093 × 10^+3^	1.728 × 10^+3^	1.134 × 10^+3^	2.311 × 10^+3^	**1.063 × 10^+3^**
	Mean	2.041 × 10^+3^	8.369 × 10^+3^	9.110 × 10^+3^	3.419 × 10^+3^	1.315 × 10^+4^	3.403 × 10^+3^	2.499 × 10^+3^	3.154 × 10^+3^	2.279 × 10^+3^	1.693 × 10^+3^	2.698 × 10^+3^	**1.168 × 10^+3^**
	Std	1.869 × 10^+2^	4.707 × 10^+2^	3.408 × 10^+2^	1.562 × 10^+2^	5.127 × 10^+2^	1.653 × 10^+2^	5.879 × 10^+2^	**4.717 × 10^+1^**	2.671 × 10^+2^	6.036 × 10^+2^	1.724 × 10^+2^	7.186 × 10^+1^
	Rank	3	10	11	9	12	8	5	7	4	2	6	1
CEC08	Best	1.206 × 10^+3^	2.388 × 10^+3^	2.365 × 10^+3^	1.858 × 10^+3^	3.098 × 10^+3^	1.850 × 10^+3^	1.658 × 10^+3^	2.154 × 10^+3^	1.499 × 10^+3^	**1.008 × 10^+3^**	1.480 × 10^+3^	1.045 × 10^+3^
	Mean	1.387 × 10^+3^	2.645 × 10^+3^	2.452 × 10^+3^	2.106 × 10^+3^	3.464 × 10^+3^	1.989 × 10^+3^	1.989 × 10^+3^	2.199 × 10^+3^	1.669 × 10^+3^	1.616 × 10^+3^	1.618 × 10^+3^	**1.126 × 10^+3^**
	Std	7.768 × 10^+1^	1.426 × 10^+2^	4.199 × 10^+1^	1.519 × 10^+2^	1.399 × 10^+2^	8.957 × 10^+1^	1.540 × 10^+2^	**1.801 × 10^+1^**	9.639 × 10^+1^	3.752 × 10^+2^	8.558 × 10^+1^	5.226 × 10^+1^
	Rank	2	11	10	8	12	6	7	9	5	3	4	1
CEC09	Best	1.408 × 10^+4^	1.047 × 10^+5^	1.084 × 10^+5^	3.627 × 10^+4^	1.723 × 10^+5^	3.235 × 10^+4^	2.093 × 10^+4^	5.267 × 10^+4^	1.975 × 10^+4^	**9.125 × 10^+2^**	2.039 × 10^+4^	3.051 × 10^+3^
	Mean	2.272 × 10^+4^	1.328 × 10^+5^	1.343 × 10^+5^	5.837 × 10^+4^	2.185 × 10^+5^	4.047 × 10^+4^	4.903 × 10^+4^	5.619 × 10^+4^	4.337 × 10^+4^	2.369 × 10^+4^	2.637 × 10^+4^	**8.251 × 10^+3^**
	Std	3.392 × 10^+3^	1.316 × 10^+4^	9.405 × 10^+3^	1.641 × 10^+4^	1.638 × 10^+4^	4.906 × 10^+3^	1.926 × 10^+4^	**2.227 × 10^+3^**	2.442 × 10^+4^	3.010 × 10^+4^	3.725 × 10^+3^	3.267 × 10^+3^
	Rank	2	10	11	8	12	6	7	9	5	3	4	1
CEC10	Best	1.280 × 10^+4^	3.161 × 10^+4^	2.841 × 10^+4^	2.081 × 10^+4^	3.419 × 10^+4^	1.825 × 10^+4^	1.268 × 10^+4^	2.876 × 10^+4^	3.846 × 10^+4^	1.841 × 10^+4^	1.597 × 10^+4^	**1.232 × 10^+4^**
	Mean	1.712 × 10^+4^	3.243 × 10^+4^	2.931 × 10^+4^	2.585 × 10^+4^	3.600 × 10^+4^	2.295 × 10^+4^	1.794 × 10^+4^	3.010 × 10^+4^	3.846 × 10^+4^	2.795 × 10^+4^	2.135 × 10^+4^	**1.615 × 10^+4^**
	Std	3.252 × 10^+3^	4.542 × 10^+2^	3.760 × 10^+2^	2.660 × 10^+3^	7.878 × 10^+2^	2.206 × 10^+3^	1.671 × 10^+3^	4.993 × 10^+2^	**2.220 × 10^−11^**	5.288 × 10^+3^	3.299 × 10^+3^	2.590 × 10^+3^
	Rank	2	10	8	6	11	5	3	9	12	7	4	1
CEC11	Best	4.326 × 10^+3^	1.619 × 10^+5^	1.309 × 10^+5^	3.089 × 10^+4^	4.148 × 10^+5^	1.446 × 10^+4^	2.884 × 10^+4^	8.732 × 10^+4^	7.063 × 10^+4^	7.525 × 10^+3^	**2.882 × 10^+3^**	3.058 × 10^+3^
	Mean	6.248 × 10^+3^	2.469 × 10^+5^	1.795 × 10^+5^	8.670 × 10^+4^	2.633 × 10^+7^	2.475 × 10^+4^	9.970 × 10^+4^	9.384 × 10^+4^	2.097 × 10^+5^	1.087 × 10^+5^	4.575 × 10^+3^	**4.352 × 10^+3^**
	Std	1.986 × 10^+3^	6.108 × 10^+4^	2.083 × 10^+4^	5.175 × 10^+4^	7.664 × 10^+7^	6.078 × 10^+3^	3.971 × 10^+4^	3.974 × 10^+3^	1.019 × 10^+5^	8.240 × 10^+4^	**1.101 × 10^+3^**	1.314 × 10^+3^
	Rank	3	11	10	5	12	4	8	6	9	7	2	1
CEC12	Best	1.006 × 10^+7^	5.639 × 10^+10^	3.400 × 10^+10^	6.826 × 10^+8^	2.606 × 10^+11^	1.145 × 10^+9^	4.683 × 10^+8^	8.315 × 10^+10^	2.958 × 10^+9^	4.379 × 10^+7^	2.034 × 10^+7^	**5.574 × 10^+6^**
	Mean	3.478 × 10^+7^	8.357 × 10^+10^	4.208 × 10^+10^	3.074 × 10^+9^	3.382 × 10^+11^	3.716 × 10^+9^	1.473 × 10^+9^	9.473 × 10^+10^	1.659 × 10^+10^	5.508 × 10^+9^	9.615 × 10^+7^	**1.394 × 10^+7^**
	Std	1.678 × 10^+7^	1.551 × 10^+10^	3.761 × 10^+9^	1.817 × 10^+9^	3.713 × 10^+10^	1.338 × 10^+9^	7.689 × 10^+8^	4.349 × 10^+9^	1.012 × 10^+10^	1.772 × 10^+10^	1.032 × 10^+8^	**6.024 × 10^+6^**
	Rank	2	10	9	6	12	7	5	11	8	4	3	1
CEC13	Best	1.513 × 10^+4^	7.198 × 10^+9^	1.036 × 10^+9^	2.575 × 10^+6^	7.090 × 10^+10^	4.193 × 10^+6^	1.568 × 10^+5^	1.615 × 10^+10^	6.455 × 10^+6^	**1.868 × 10^+3^**	4.061 × 10^+4^	2.857 × 10^+3^
	Mean	2.957 × 10^+4^	1.380 × 10^+10^	1.462 × 10^+9^	7.742 × 10^+6^	8.886 × 10^+10^	1.659 × 10^+8^	7.644 × 10^+7^	1.885 × 10^+10^	3.121 × 10^+9^	6.916 × 10^+6^	1.320 × 10^+5^	**8.822 × 10^+3^**
	Std	9.707 × 10^+3^	3.383 × 10^+9^	1.698 × 10^+8^	6.456 × 10^+6^	9.725 × 10^+9^	2.077 × 10^+8^	9.377 × 10^+7^	1.352 × 10^+9^	3.420 × 10^+9^	3.370 × 10^+7^	1.112 × 10^+5^	**6.487 × 10^+3^**
	Rank	2	10	8	5	12	7	6	11	9	3	4	1
CEC14	Best	**6.923 × 10^+4^**	1.579 × 10^+7^	1.954 × 10^+7^	1.320 × 10^+6^	1.796 × 10^+8^	1.706 × 10^+6^	2.248 × 10^+6^	1.015 × 10^+7^	2.695 × 10^+6^	8.126 × 10^+4^	7.349 × 10^+4^	7.685 × 10^+4^
	Mean	5.361 × 10^+5^	5.457 × 10^+7^	4.019 × 10^+7^	7.028 × 10^+6^	6.765 × 10^+8^	4.499 × 10^+6^	7.934 × 10^+6^	1.592 × 10^+7^	1.274 × 10^+7^	1.030 × 10^+7^	**2.054 × 10^+5^**	3.550 × 10^+5^
	Std	2.368 × 10^+5^	2.300 × 10^+7^	1.035 × 10^+7^	5.017 × 10^+6^	2.714 × 10^+8^	1.817 × 10^+6^	5.020 × 10^+6^	2.269 × 10^+6^	8.366 × 10^+6^	8.426 × 10^+6^	**1.406 × 10^+5^**	1.801 × 10^+5^
	Rank	3	11	10	5	12	4	6	9	8	7	1	2
CEC15	Best	3.560 × 10^+3^	2.437 × 10^+9^	1.078 × 10^+8^	2.397 × 10^+5^	3.599 × 10^+10^	7.263 × 10^+4^	4.477 × 10^+4^	5.942 × 10^+9^	5.583 × 10^+4^	**1.809 × 10^+3^**	1.640 × 10^+4^	1.904 × 10^+3^
	Mean	1.015 × 10^+4^	5.356 × 10^+9^	1.854 × 10^+8^	9.925 × 10^+5^	4.253 × 10^+10^	1.500 × 10^+7^	8.312 × 10^+6^	7.069 × 10^+9^	2.065 × 10^+9^	1.549 × 10^+7^	9.354 × 10^+4^	**4.952 × 10^+3^**
	Std	7.725 × 10^+3^	1.723 × 10^+9^	4.443 × 10^+7^	7.510 × 10^+5^	5.405 × 10^+09^	2.088 × 10^+7^	2.723 × 10^+7^	5.516 × 10^+8^	2.679 × 10^+9^	5.771 × 10^+7^	1.261 × 10^+5^	**5.687 × 10^+3^**
	Rank	2	10	8	6	12	7	5	11	9	3	4	1
CEC16	Best	4.498 × 10^+3^	1.184 × 10^+4^	1.090 × 10^+4^	1.076 × 10^+4^	2.570 × 10^+4^	7.612 × 10^+3^	6.624 × 10^+3^	1.302 × 10^+4^	7.366 × 10^+3^	7.077 × 10^+3^	5.275 × 10^+3^	**4.121 × 10^+3^**
	Mean	5.761 × 10^+3^	1.330 × 10^+4^	1.183 × 10^+4^	1.397 × 10^+4^	3.656 × 10^+4^	1.034 × 10^+4^	8.173 × 10^+3^	1.401 × 10^+4^	9.376 × 10^+3^	9.750 × 10^+3^	7.314 × 10^+3^	**5.522 × 10^+3^**
	Std	5.936 × 10^+2^	8.210 × 10^+2^	4.327 × 10^+2^	1.728 × 10^+3^	5.237 × 10^+3^	1.126 × 10^+3^	1.027 × 10^+3^	**3.683 × 10^+2^**	1.294 × 10^+3^	1.144 × 10^+3^	1.519 × 10^+3^	7.987 × 10^+2^
	Rank	2	9	8	10	12	7	4	11	5	6	3	1
CEC17	Best	3.919 × 10^+3^	1.312 × 10^+4^	8.541 × 10^+3^	6.004 × 10^+3^	1.210 × 10^+7^	5.488 × 10^+3^	5.643 × 10^+3^	1.494 × 10^+4^	6.877 × 10^+3^	6.041 × 10^+3^	5.280 × 10^+3^	**3.459 × 10^+3^**
	Mean	5.021 × 10^+3^	5.133 × 10^+4^	9.477 × 10^+3^	7.875 × 10^+3^	6.376 × 10^+7^	7.579 × 10^+3^	7.900 × 10^+3^	2.767 × 10^+4^	9.458 × 10^+4^	8.250 × 10^+3^	6.410 × 10^+3^	**4.822 × 10^+3^**
	Std	5.535 × 10^+2^	8.347 × 10^+4^	**4.699 × 10^+2^**	9.812 × 10^+2^	3.607 × 10^+7^	1.691 × 10^+3^	9.708 × 10^+2^	5.655 × 10^+3^	4.140 × 10^+5^	3.391 × 10^+3^	6.239 × 10^+2^	6.304 × 10^+2^
	Rank	2	10	9	6	12	4	7	11	8	5	3	1
CEC18	Best	3.734 × 10^+5^	4.288 × 10^+7^	3.362 × 10^+7^	1.219 × 10^+6^	5.789 × 10^+8^	1.780 × 10^+6^	3.247 × 10^+6^	1.311 × 10^+7^	5.792 × 10^+6^	9.811 × 10^+5^	**2.679 × 10^+5^**	4.071 × 10^+5^
	Mean	1.168 × 10^+6^	9.027 × 10^+7^	5.991 × 10^+7^	7.124 × 10^+6^	1.322 × 10^+9^	3.909 × 10^+6^	1.124 × 10^+7^	2.250 × 10^+7^	3.641 × 10^+7^	2.350 × 10^+7^	**5.194 × 10^+5^**	8.226 × 10^+5^
	Std	5.423 × 10^+5^	3.369 × 10^+7^	1.412 × 10^+7^	4.328 × 10^+6^	4.436 × 10^+8^	1.356 × 10^+6^	7.402 × 10^+6^	3.874 × 10^+6^	2.992 × 10^+7^	4.006 × 10^+7^	**1.937 × 10^+5^**	3.368 × 10^+5^
	Rank	3	11	10	5	12	4	6	8	9	7	1	2
CEC19	Best	2.362 × 10^+3^	1.525 × 10^+9^	1.706 × 10^+8^	3.862 × 10^+6^	2.891 × 10^+10^	7.189 × 10^+6^	5.677 × 10^+4^	4.994 × 10^+9^	3.456 × 10^+6^	**2.020 × 10^+3^**	2.718 × 10^+4^	2.097 × 10^+3^
	Mean	9.591 × 10^+3^	4.438 × 10^+9^	3.217 × 10^+8^	3.166 × 10^+7^	4.353 × 10^+10^	5.270 × 10^+7^	2.407 × 10^+7^	6.136 × 10^+9^	6.314 × 10^+8^	6.601 × 10^+5^	1.385 × 10^+5^	**5.417 × 10^+3^**
	Std	9.660 × 10^+3^	1.617 × 10^+9^	6.736 × 10^+7^	2.212 × 10^+7^	6.647 × 10^+9^	1.164 × 10^+8^	2.388 × 10^+7^	5.959 × 10^+8^	1.027 × 10^+9^	1.812 × 10^+6^	1.410 × 10^+5^	**4.125 × 10^+3^**
	Rank	2	10	9	6	12	7	5	11	8	3	4	1
CEC20	Best	4.117 × 10^+3^	7.100 × 10^+3^	5.899 × 10^+3^	5.499 × 10^+3^	8.324 × 10^+3^	5.067 × 10^+3^	4.992 × 10^+3^	6.363 × 10^+3^	7.052 × 10^+3^	5.639 × 10^+3^	4.752 × 10^+3^	**3.947 × 10^+3^**
	Mean	5.187 × 10^+3^	7.888 × 10^+3^	6.786 × 10^+3^	6.691 × 10^+3^	9.660 × 10^+3^	6.076 × 10^+3^	6.062 × 10^+3^	6.785 × 10^+3^	8.275 × 10^+3^	6.631 × 10^+3^	5.760 × 10^+3^	**4.939 × 10^+3^**
	Std	5.829 × 10^+2^	2.833 × 10^+2^	3.409 × 10^+2^	6.119 × 10^+2^	3.967 × 10^+2^	5.381 × 10^+2^	6.682 × 10^+2^	**2.173 × 10^+2^**	5.791 × 10^+2^	9.393 × 10^+2^	4.640 × 10^+2^	5.212 × 10^+2^
	Rank	2	10	9	7	12	4	5	8	11	6	3	1
CEC21	Best	2.695 × 10^+3^	4.050 × 10^+3^	3.874 × 10^+3^	3.765 × 10^+3^	5.034 × 10^+3^	3.390 × 10^+3^	3.371 × 10^+3^	3.803 × 10^+3^	3.555 × 10^+3^	2.638 × 10^+3^	3.166 × 10^+3^	**2.510 × 10^+3^**
	Mean	2.843 × 10^+3^	4.281 × 10^+3^	4.022 × 10^+3^	4.314 × 10^+3^	5.345 × 10^+3^	3.690 × 10^+3^	3.572 × 10^+3^	3.860 × 10^+3^	4.177 × 10^+3^	3.081 × 10^+3^	3.392 × 10^+3^	**2.606 × 10^+3^**
	Std	8.851 × 10^+1^	1.453 × 10^+2^	5.202 × 10^+1^	2.531 × 10^+2^	1.687 × 10^+2^	1.736 × 10^+2^	1.121 × 10^+2^	**3.111 × 10^+1^**	3.462 × 10^+2^	3.466 × 10^+2^	1.350 × 10^+2^	8.632 × 10^+1^
	Rank	2	11	8	10	12	6	5	7	9	3	4	1
CEC22	Best	**1.566 × 10^+4^**	3.278 × 10^+4^	3.015 × 10^+4^	2.311 × 10^+4^	3.756 × 10^+4^	2.226 × 10^+4^	1.787 × 10^+4^	3.156 × 10^+4^	2.488 × 10^+4^	1.939 × 10^+4^	1.804 × 10^+4^	1.687 × 10^+4^
	Mean	2.155 × 10^+4^	3.425 × 10^+4^	3.116 × 10^+4^	2.974 × 10^+4^	3.879 × 10^+4^	2.577 × 10^+4^	2.089 × 10^+4^	3.267 × 10^+4^	2.816 × 10^+4^	2.894 × 10^+4^	2.404 × 10^+4^	**1.973 × 10^+4^**
	Std	3.087 × 10^+3^	6.017 × 10^+2^	**4.286 × 10^+2^**	3.140 × 10^+3^	5.745 × 10^+2^	1.833 × 10^+3^	1.862 × 10^+3^	4.966 × 10^+2^	1.942 × 10^+3^	4.764 × 10^+3^	2.718 × 10^+3^	2.085 × 10^+3^
	Rank	3	11	9	8	12	5	2	10	6	7	4	1
CEC23	Best	3.258 × 10^+3^	4.605 × 10^+3^	3.927 × 10^+3^	4.754 × 10^+3^	7.376 × 10^+3^	4.276 × 10^+3^	3.892 × 10^+3^	5.625 × 10^+3^	8.724 × 10^+3^	3.019 × 10^+3^	3.833 × 10^+3^	**3.016 × 10^+3^**
	Mean	3.441 × 10^+3^	5.011 × 10^+3^	3.993 × 10^+3^	5.337 × 10^+3^	8.159 × 10^+3^	4.568 × 10^+3^	4.219 × 10^+3^	5.792 × 10^+3^	8.724 × 10^+3^	3.494 × 10^+3^	4.468 × 10^+3^	**3.161 × 10^+3^**
	Std	1.064 × 10^+2^	2.425 × 10^+2^	2.209 × 10^+1^	3.041 × 10^+2^	4.690 × 10^+2^	1.744 × 10^+2^	1.514 × 10^+2^	7.152 × 10^+1^	**3.700 × 10^−12^**	5.017 × 10^+2^	3.579 × 10^+2^	6.781 × 10^+1^
	Rank	3	8	4	9	11	7	5	10	12	2	6	1
CEC24	Best	3.664 × 10^+3^	5.194 × 10^+3^	4.461 × 10^+3^	5.694 × 10^+3^	1.160 × 10^+4^	4.938 × 10^+3^	4.526 × 10^+3^	7.587 × 10^+3^	1.423 × 10^+4^	**3.461 × 10^+3^**	4.580 × 10^+3^	3.539 × 10^+3^
	Mean	4.124 × 10^+3^	5.979 × 10^+3^	4.588 × 10^+3^	6.951 × 10^+3^	1.376 × 10^+4^	5.811 × 10^+3^	5.027 × 10^+3^	7.950 × 10^+3^	1.423 × 10^+4^	4.054 × 10^+3^	5.707 × 10^+3^	**3.637 × 10^+3^**
	Std	4.932 × 10^+2^	4.214 × 10^+2^	3.501 × 10^+1^	6.592 × 10^+2^	9.340 × 10^+2^	3.934 × 10^+2^	2.571 × 10^+2^	1.489 × 10^+2^	**7.400 × 10^−12^**	4.475 × 10^+2^	7.148 × 10^+2^	5.968 × 10^+1^
	Rank	3	8	4	9	11	7	5	10	12	2	6	1
CEC25	Best	3.409 × 10^+3^	3.893 × 10^+4^	4.672 × 10^+4^	4.294 × 10^+3^	8.876 × 10^+4^	5.121 × 10^+3^	3.540 × 10^+3^	1.421 × 10^+4^	3.589 × 10^+3^	3.270 × 10^+3^	3.478 × 10^+3^	**3.251 × 10^+3^**
	Mean	3.612 × 10^+3^	5.584 × 10^+4^	5.561 × 10^+4^	4.678 × 10^+3^	1.256 × 10^+5^	5.888 × 10^+3^	8.259 × 10^+3^	1.489 × 10^+4^	4.368 × 10^+3^	6.534 × 10^+3^	3.630 × 10^+3^	**3.449 × 10^+3^**
	Std	8.397 × 10^+1^	7.960 × 10^+3^	4.866 × 10^+3^	3.824 × 10^+2^	1.773 × 10^+4^	4.766 × 10^+2^	4.612 × 10^+3^	2.262 × 10^+2^	5.622 × 10^+2^	1.073 × 10^+4^	9.013 × 10^+1^	**6.705 × 10^+1^**
	Rank	2	11	10	6	12	8	7	9	5	3	4	1
CEC26	Best	1.078 × 10^+4^	2.577 × 10^+4^	1.954 × 10^+4^	2.784 × 10^+4^	7.036 × 10^+4^	2.575 × 10^+4^	2.006 × 10^+4^	3.117 × 10^+4^	8.882 × 10^+4^	8.331 × 10^+3^	1.659 × 10^+4^	**3.647 × 10^+3^**
	Mean	1.293 × 10^+4^	3.006 × 10^+4^	2.025 × 10^+4^	3.515 × 10^+4^	8.711 × 10^+4^	3.106 × 10^+4^	2.399 × 10^+4^	3.191 × 10^+4^	8.882 × 10^+4^	1.402 × 10^+4^	2.171 × 10^+4^	**9.067 × 10^+3^**
	Std	1.750 × 10^+3^	2.251 × 10^+3^	3.653 × 10^+2^	3.922 × 10^+3^	8.875 × 10^+3^	2.892 × 10^+3^	2.365 × 10^+3^	4.159 × 10^+2^	**0.000 × 10^+0^**	6.779 × 10^+3^	3.224 × 10^+3^	1.843 × 10^+3^
	Rank	2	7	4	10	11	8	6	9	12	3	5	1
CEC27	Best	3.561 × 10^+3^	4.416 × 10^+3^	3.980 × 10^+3^	3.200 × 10^+3^	1.427 × 10^+4^	4.042 × 10^+3^	3.579 × 10^+3^	7.453 × 10^+3^	3.840 × 10^+3^	**3.200 × 10^+3^**	3.760 × 10^+3^	3.439 × 10^+3^
	Mean	4.087 × 10^+3^	5.181 × 10^+3^	4.188 × 10^+3^	3.200 × 10^+3^	1.667 × 10^+4^	4.596 × 10^+3^	4.015 × 10^+3^	7.922 × 10^+3^	4.419 × 10^+3^	**3.200 × 10^+3^**	4.418 × 10^+3^	3.684 × 10^+3^
	Std	5.506 × 10^+2^	5.009 × 10^+2^	7.984 × 10^+1^	**2.477 × 10^−4^**	1.417 × 10^+3^	4.180 × 10^+2^	2.380 × 10^+2^	2.039 × 10^+2^	3.514 × 10^+2^	4.285 × 10^−4^	4.331 × 10^+2^	2.093 × 10^+2^
	Rank	5	10	6	1	12	9	4	11	8	2	7	3
CEC28	Best	3.538 × 10^+3^	2.091 × 10^+4^	1.617 × 10^+4^	3.300 × 10^+3^	5.453 × 10^+4^	4.484 × 10^+3^	5.157 × 10^+3^	1.489 × 10^+4^	4.015 × 10^+3^	3.300 × 10^+3^	3.488 × 10^+3^	3.487 × 10^+3^
	Mean	3.736 × 10^+3^	2.866 × 10^+4^	1.647 × 10^+4^	3.300 × 10^+3^	6.936 × 10^+4^	6.167 × 10^+3^	1.719 × 10^+4^	1.550 × 10^+4^	6.229 × 10^+3^	3.300 × 10^+3^	3.636 × 10^+3^	3.587 × 10^+3^
	Std	1.198 × 10^+2^	6.186 × 10^+3^	1.270 × 10^+2^	**2.516 × 10^−4^**	7.119 × 10^+3^	7.805 × 10^+2^	5.521 × 10^+3^	2.975 × 10^+2^	2.017 × 10^+3^	5.236 × 10^−4^	6.696 × 10^+1^	4.447 × 10^+1^
	Rank	5	11	9	2	12	7	10	8	6	1	4	3
CEC29	Best	5.485 × 10^+3^	1.727 × 10^+4^	1.116 × 10^+4^	7.946 × 10^+3^	2.209 × 10^+6^	1.022 × 10^+4^	8.291 × 10^+3^	2.341 × 10^+4^	7.287 × 10^+3^	5.980 × 10^+3^	7.787 × 10^+3^	**5.005 × 10^+3^**
	Mean	6.779 × 10^+3^	3.155 × 10^+4^	1.229 × 10^+4^	1.448 × 10^+4^	1.418 × 10^+7^	1.393 × 10^+4^	1.013 × 10^+4^	2.894 × 10^+4^	1.970 × 10^+4^	9.354 × 10^+3^	1.089 × 10^+4^	**6.361 × 10^+3^**
	Std	6.611 × 10^+2^	8.819 × 10^+3^	**5.543 × 10^+2^**	2.800 × 10^+3^	9.130 × 10^+6^	1.752 × 10^+3^	9.695 × 10^+2^	3.487 × 10^+3^	1.561 × 10^+4^	2.806 × 10^+3^	1.386 × 10^+3^	5.860 × 10^+2^
	Rank	2	11	6	8	12	7	4	10	9	3	5	1
CEC30	Best	6.433 × 10^+4^	3.418 × 10^+9^	1.153 × 10^+8^	4.595 × 10^+7^	4.448 × 10^+10^	1.801 × 10^+8^	2.918 × 10^+6^	1.308 × 10^+10^	6.334 × 10^+7^	**3.420 × 10^+3^**	5.587 × 10^+5^	1.033 × 10^+4^
	Mean	3.317 × 10^+5^	7.402 × 10^+9^	1.671 × 10^+8^	4.141 × 10^+8^	6.986 × 10^+10^	4.720 × 10^+8^	5.730 × 10^+7^	1.624 × 10^+10^	2.351 × 10^+9^	9.830 × 10^+6^	3.166 × 10^+6^	**2.273 × 10^+4^**
	Std	4.408 × 10^+5^	1.750 × 10^+9^	3.250 × 10^+7^	3.968 × 10^+8^	1.159 × 10^+10^	1.889 × 10^+8^	8.123 × 10^+7^	1.298 × 10^+9^	1.550 × 10^+9^	4.552 × 10^+7^	4.420 × 10^+6^	**9.291 × 10^+3^**
	Rank	3	10	6	7	12	8	5	11	9	2	4	1
Mean Rank		2.69	10.17	8.34	6.7	11.86	6.28	5.66	9.24	8.03	3.86	3.86	1.27
Final Ranking		2	11	9	7	12	6	5	10	8	3	3	1

**Table 5:** Wilcoxon rank-sum test (Dim = 100).

Function	HBA	PSO	DE	WOA	AOA	PO	DBO	GQPSO	QHDBO	LSHADE	SaCHBA_PDN
CEC01	9.063 × 10^−8^	3.019 × 10^−11^	3.019 × 10^−11^	3.019 × 10^−11^	3.019 × 10^−11^	3.019 × 10^−11^	3.019 × 10^−11^	3.019 × 10^−11^	3.019 × 10^−11^	8.120 × 10^−4^	3.019 × 10^−11^
	+	+	+	+	+	+	+	+	+	+	+
CEC03	3.965 × 10^−8^	3.019 × 10^−11^	3.019 × 10^−11^	3.019 × 10^−11^	3.019 × 10^−11^	3.019 × 10^−11^	3.019 × 10^−11^	3.019 × 10^−11^	3.019 × 10^−11^	1.784 × 10^−4^	3.019 × 10^−11^
	+	+	+	+	+	+	+	+	+	+	+
CEC04	2.879 × 10^−6^	3.019 × 10^−11^	3.019 × 10^−11^	3.019 × 10^−11^	3.019 × 10^−11^	3.019 × 10^−11^	3.019 × 10^−11^	3.019 × 10^−11^	3.019 × 10^−11^	2.006 × 10^−4^	3.019 × 10^−11^
	+	+	+	+	+	+	+	+	+	+	+
CEC05	4.998 × 10^−9^	3.019 × 10^−11^	3.019 × 10^−11^	3.019 × 10^−11^	3.019 × 10^−11^	3.019 × 10^−11^	3.019 × 10^−11^	3.019 × 10^−11^	3.019 × 10^−11^	2.068 × 10^−2^	3.019 × 10^−11^
	+	+	+	+	+	+	+	+	+	+	+
CEC06	4.998 × 10^−9^	3.019 × 10^−11^	3.019 × 10^−11^	3.019 × 10^−11^	3.019 × 10^−11^	3.019 × 10^−11^	3.019 × 10^−11^	3.019 × 10^−11^	3.019 × 10^−11^	3.006 × 10^−4^	3.019 × 10^−11^
	+	+	+	+	+	+	+	+	+	+	+
CEC07	5.186 × 10^−7^	3.019 × 10^−11^	3.019 × 10^−11^	3.019 × 10^−11^	3.019 × 10^−11^	3.019 × 10^−11^	3.019 × 10^−11^	3.019 × 10^−11^	3.019 × 10^−11^	1.114 × 10^−3^	3.019 × 10^−11^
	+	+	+	+	+	+	+	+	+	+	+
CEC08	1.492 × 10^−6^	3.019 × 10^−11^	3.019 × 10^−11^	3.019 × 10^−11^	3.019 × 10^−11^	3.019 × 10^−11^	3.019 × 10^−11^	3.019 × 10^−11^	3.019 × 10^−11^	8.197 × 10^−7^	3.019 × 10^−11^
	+	+	+	+	+	+	+	+	+	+	+
CEC09	3.094 × 10^−6^	3.019 × 10^−11^	3.019 × 10^−11^	3.019 × 10^−11^	3.019 × 10^−11^	3.019 × 10^−11^	3.019 × 10^−11^	3.019 × 10^−11^	3.019 × 10^−11^	4.982 × 10^−4^	3.019 × 10^−11^
	+	+	+	+	+	+	+	+	+	+	+
CEC10	5.462 × 10^−9^	3.019 × 10^−11^	3.019 × 10^−11^	3.019 × 10^−11^	3.019 × 10^−11^	3.019 × 10^−11^	3.019 × 10^−11^	3.019 × 10^−11^	3.019 × 10^−11^	5.874 × 10^−4^	3.019 × 10^−11^
	+	+	+	+	+	+	+	+	+	+	+
CEC11	4.801 × 10^−7^	3.019 × 10^−11^	3.019 × 10^−11^	3.019 × 10^−11^	3.019 × 10^−11^	3.019 × 10^−11^	3.019 × 10^−11^	3.019 × 10^−11^	3.019 × 10^−11^	4.856 × 10^−3^	3.019 × 10^−11^
	+	+	+	+	+	+	+	+	+	+	+
CEC12	3.646 × 10^−8^	3.019 × 10^−11^	3.019 × 10^−11^	3.019 × 10^−11^	3.019 × 10^−11^	3.019 × 10^−11^	3.019 × 10^−11^	3.019 × 10^−11^	3.019 × 10^−11^	3.835 × 10^−6^	3.019 × 10^−11^
	+	+	+	+	+	+	+	+	+	+	+
CEC13	6.518 × 10^−9^	3.019 × 10^−11^	3.019 × 10^−11^	3.019 × 10^−11^	3.019 × 10^−11^	3.019 × 10^−11^	3.019 × 10^−11^	3.019 × 10^−11^	3.019 × 10^−11^	3.006 × 10^−4^	3.019 × 10^−11^
	+	+	+	+	+	+	+	+	+	+	+
CEC14	7.043 × 10^−7^	3.019 × 10^−11^	3.019 × 10^−11^	3.019 × 10^−11^	3.019 × 10^−11^	3.019 × 10^−11^	3.019 × 10^−11^	3.019 × 10^−11^	4.975 × 10^−11^	2.531 × 10^−4^	3.019 × 10^−11^
	+	+	+	+	+	+	+	+	+	+	+
CEC15	5.600 × 10^−7^	3.019 × 10^−11^	3.019 × 10^−11^	3.019 × 10^−11^	3.019 × 10^−11^	3.019 × 10^−11^	3.019 × 10^−11^	3.019 × 10^−11^	3.019 × 10^−11^	2.709 × 10^−2^	3.019 × 10^−11^
	+	+	+	+	+	+	+	+	+	+	+
CEC16	4.311 × 10^−8^	3.019 × 10^−11^	3.019 × 10^−11^	3.019 × 10^−11^	3.019 × 10^−11^	3.019 × 10^−11^	3.019 × 10^−11^	3.019 × 10^−11^	3.019 × 10^−11^	1.996 × 10^−5^	3.019 × 10^−11^
	+	+	+	+	+	+	+	+	+	+	+
CEC17	2.195 × 10^−8^	3.019 × 10^−11^	3.019 × 10^−11^	3.019 × 10^−11^	3.019 × 10^−11^	3.019 × 10^−11^	3.019 × 10^−11^	3.019 × 10^−11^	3.019 × 10^−11^	4.060 × 10^−2^	3.019 × 10^−11^
	+	+	+	+	+	+	+	+	+	+	+
CEC18	1.102 × 10^−8^	3.019 × 10^−11^	3.019 × 10^−11^	3.019 × 10^−11^	3.019 × 10^−11^	3.019 × 10^−11^	3.019 × 10^−11^	3.019 × 10^−11^	3.019 × 10^−11^	8.564 × 10^−4^	3.019 × 10^−11^
	+	+	+	+	+	+	+	+	+	+	+
CEC19	1.850 × 10^−8^	3.019 × 10^−11^	3.019 × 10^−11^	3.019 × 10^−11^	3.019 × 10^−11^	3.019 × 10^−11^	3.019 × 10^−11^	3.019 × 10^−11^	3.019 × 10^−11^	2.278 × 10^−5^	3.019 × 10^−11^
	+	+	+	+	+	+	+	+	+	+	+
CEC20	4.444 × 10^−7^	3.019 × 10^−11^	3.019 × 10^−11^	3.019 × 10^−11^	3.019 × 10^−11^	3.019 × 10^−11^	3.019 × 10^−11^	3.019 × 10^−11^	3.019 × 10^−11^	2.891 × 10^−3^	3.019 × 10^−11^
	+	+	+	+	+	+	+	+	+	+	+
CEC21	9.063 × 10^−8^	3.019 × 10^−11^	3.019 × 10^−11^	3.019 × 10^−11^	3.019 × 10^−11^	3.019 × 10^−11^	3.019 × 10^−11^	3.019 × 10^−11^	3.019 × 10^−11^	6.913 × 10^−4^	3.019 × 10^−11^
	+	+	+	+	+	+	+	+	+	+	+
CEC22	5.092 × 10^−8^	3.019 × 10^−11^	3.019 × 10^−11^	3.019 × 10^−11^	3.019 × 10^−11^	3.019 × 10^−11^	3.019 × 10^−11^	3.019 × 10^−11^	1.777 × 10^−10^	8.292 × 10^−6^	3.019 × 10^−11^
	+	+	+	+	+	+	+	+	+	+	+
CEC23	3.646 × 10^−08^	3.019 × 10^−11^	3.019 × 10^−11^	3.019 × 10^−11^	3.019 × 10^−11^	3.019 × 10^−11^	3.019 × 10^−11^	3.019 × 10^−11^	3.019 × 10^−11^	1.058 × 10^−3^	3.019 × 10^−11^
	+	+	+	+	+	+	+	+	+	+	+
CEC24	3.081 × 10^−08^	3.019 × 10^−11^	3.019 × 10^−11^	3.019 × 10^−11^	3.019 × 10^−11^	3.019 × 10^−11^	3.019 × 10^−11^	3.019 × 10^−11^	3.019 × 10^−11^	8.771 × 10^−2^	3.019 × 10^−11^
	+	+	+	+	+	+	+	+	+	-	+
CEC25	1.206 × 10^−10^	3.002 × 10^−11^	3.019 × 10^−11^	3.019 × 10^−11^	3.019 × 10^−11^	3.019 × 10^−11^	3.019 × 10^−11^	3.019 × 10^−11^	3.019 × 10^−11^	5.091 × 10^−6^	3.019 × 10^−11^
	+	+	+	+	+	+	+	+	+	+	+
CEC26	2.390 × 10^−08^	3.019 × 10^−11^	3.019 × 10^−11^	3.019 × 10^−11^	3.019 × 10^−11^	3.019 × 10^−11^	3.019 × 10^−11^	3.019 × 10^−11^	3.019 × 10^−11^	2.510 × 10^−2^	3.019 × 10^−11^
	+	+	+	+	+	+	+	+	+	+	+
CEC27	3.646 × 10^−08^	3.019 × 10^−11^	3.019 × 10^−11^	3.019 × 10^−11^	3.019 × 10^−11^	3.019 × 10^−11^	3.019 × 10^−11^	3.019 × 10^−11^	3.019 × 10^−11^	5.943 × 10^−2^	3.019 × 10^−11^
	+	+	+	+	+	+	+	+	+	-	+
CEC28	2.602 × 10^−08^	3.019 × 10^−11^	3.019 × 10^−11^	3.019 × 10^−11^	3.019 × 10^−11^	3.019 × 10^−11^	3.019 × 10^−11^	3.019 × 10^−11^	3.019 × 10^−11^	4.353 × 10^−5^	3.019 × 10^−11^
	+	+	+	+	+	+	+	+	+	+	+
CEC29	6.010 × 10^−08^	3.019 × 10^−11^	3.019 × 10^−11^	3.019 × 10^−11^	3.019 × 10^−11^	3.019 × 10^−11^	3.019 × 10^−11^	3.019 × 10^−11^	3.019 × 10^−11^	4.226 × 10^−3^	3.019 × 10^−11^
	+	+	+	+	+	+	+	+	+	+	+
CEC30	6.528 × 10^−08^	3.019 × 10^−11^	3.019 × 10^−11^	3.019 × 10^−11^	3.019 × 10^−11^	3.019 × 10^−11^	3.019 × 10^−11^	3.019 × 10^−11^	3.019 × 10^−11^	2.002 × 10^−6^	3.019 × 10^−11^
	+	+	+	+	+	+	+	+	+	+	+
+/=/-	29/0/0	29/0/0	29/0/0	29/0/0	29/0/0	29/0/0	29/0/0	29/0/0	29/0/0	27/0/2	29/0/0

**Table 6:** Results of ablation experiment.

Funciton	Index	HBA	LRMHBA1	LRMHBA2	LRMHBA3	LRMHBA
CEC01	Best	**1.012 × 10^+2^**	1.013 × 10^+2^	1.025 × 10^+2^	1.190 × 10^+2^	1.150 × 10^+2^
	Mean	6.561 × 10^+3^	5.258 × 10^+3^	3.687 × 10^+3^	5.393 × 10^+3^	**3.302 × 10^+3^**
	Std	7.134 × 10^+3^	5.978 × 10^+3^	**3.596 × 10^+3^**	4.856 × 10^+3^	3.827 × 10^+3^
	Rank	5	3	2	4	1
CEC03	Best	1.248 × 10^+3^	7.671 × 10^+2^	1.552 × 10^+3^	**3.036 × 10^+2^**	3.048 × 10^+2^
	Mean	3.231 × 10^+3^	3.584 × 10^+3^	4.083 × 10^+3^	**4.298 × 10^+2^**	9.780 × 10^+2^
	Std	1.327 × 10^+3^	3.115 × 10^+3^	1.529 × 10^+3^	**1.707 × 10^+2^**	1.959 × 10^+3^
	Rank	4	3	5	1	2
CEC04	Best	4.049 × 10^+2^	4.138 × 10^+2^	4.601 × 10^+2^	4.010 × 10^+2^	**4.001 × 10^+2^**
	Mean	4.818 × 10^+2^	**4.804 × 10^+2^**	4.912 × 10^+2^	4.855 × 10^+2^	4.824 × 10^+2^
	Std	2.386 × 10^+1^	2.517 × 10^+1^	**1.758 × 10^+1^**	2.070 × 10^+1^	2.875 × 10^+1^
	Rank	1	3	5	4	2
CEC05	Best	5.497 × 10^+2^	5.647 × 10^+2^	5.348 × 10^+2^	5.348 × 10^+2^	**5.229 × 10^+2^**
	Mean	6.041 × 10^+2^	6.234 × 10^+2^	5.698 × 10^+2^	5.662 × 10^+2^	**5.551 × 10^+2^**
	Std	2.948 × 10^+1^	3.347 × 10^+1^	2.041 × 10^+1^	**1.651 × 10^+1^**	1.852 × 10^+1^
	Rank	4	5	3	2	1
CEC06	Best	6.004 × 10^+2^	6.006 × 10^+2^	6.001 × 10^+2^	**6.000 × 10^+2^**	**6.000 × 10^+2^**
	Mean	6.043 × 10^+2^	6.065 × 10^+2^	6.003 × 10^+2^	**6.000 × 10^+2^**	6.080 × 10^+2^
	Std	4.665 × 10^+0^	5.925 × 10^+0^	2.303 × 10^−1^	**3.328 × 10^−3^**	3.035 × 10^+1^
	Rank	4	5	3	2	1
CEC07	Best	7.944 × 10^+2^	7.905 × 10^+2^	7.622 × 10^+2^	7.641 × 10^+2^	**7.575 × 10^+2^**
	Mean	8.575 × 10^+2^	8.722 × 10^+2^	7.957 × 10^+2^	8.021 × 10^+2^	**7.810 × 10^+2^**
	Std	3.717 × 10^+1^	4.464 × 10^+1^	1.798 × 10^+1^	2.602 × 10^+1^	**1.454 × 10^+1^**
	Rank	4	5	2	3	1
CEC08	Best	8.557 × 10^+2^	8.458 × 10^+2^	8.408 × 10^+2^	**8.368 × 10^+2^**	8.388 × 10^+2^
	Mean	8.972 × 10^+2^	8.993 × 10^+2^	8.650 × 10^+2^	8.646 × 10^+2^	**8.588 × 10^+2^**
	Std	2.153 × 10^+1^	1.991 × 10^+1^	**1.197 × 10^+1^**	1.759 × 10^+1^	1.462 × 10^+1^
	Rank	4	5	3	2	1
CEC09	Best	1.070 × 10^+3^	1.033 × 10^+3^	9.039 × 10^+2^	9.003 × 10^+2^	**9.000 × 10^+2^**
	Mean	2.049 × 10^+3^	2.426 × 10^+3^	9.460 × 10^+2^	9.943 × 10^+2^	**9.043 × 10^+2^**
	Std	6.578 × 10^+2^	8.011 × 10^+2^	4.096 × 10^+1^	2.297 × 10^+2^	**7.905 × 10^+0^**
	Rank	4	5	3	2	1
CEC10	Best	3.491 × 10^+3^	3.689 × 10^+3^	4.443 × 10^+3^	**3.174 × 10^+3^**	3.569 × 10^+3^
	Mean	4.821 × 10^+3^	5.268 × 10^+3^	5.879 × 10^+3^	**4.615 × 10^+3^**	5.079 × 10^+3^
	Std	9.057 × 10^+2^	1.204 × 10^+3^	1.053 × 10^+3^	**7.697 × 10^+2^**	8.708 × 10^+2^
	Rank	2	4	5	1	3
CEC11	Best	1.145 × 10^+3^	1.130 × 10^+3^	1.114 × 10^+3^	1.119 × 10^+3^	**1.114 × 10^+3^**
	Mean	1.226 × 10^+3^	1.215 × 10^+3^	1.178 × 10^+3^	1.147 × 10^+3^	**1.139 × 10^+3^**
	Std	4.197 × 10^+1^	4.861 × 10^+1^	4.143 × 10^+1^	2.734 × 10^+1^	**2.272 × 10^+1^**
	Rank	5	4	3	2	1
CEC12	Best	1.090 × 10^+4^	8.781 × 10^+3^	2.404 × 10^+4^	**5.149 × 10^+3^**	8.858 × 10^+3^
	Mean	7.432 × 10^+4^	8.874 × 10^+4^	9.861 × 10^+4^	**3.602 × 10^+4^**	6.223 × 10^+8^
	Std	6.303 × 10^+4^	9.746 × 10^+4^	8.322 × 10^+4^	**2.397 × 10^+4^**	3.409 × 10^+9^
	Rank	4	3	5	1	2
CEC13	Best	5.638 × 10^+3^	2.675 × 10^+3^	1.678 × 10^+3^	1.467 × 10^+3^	**1.343 × 10^+3^**
	Mean	3.419 × 10^+4^	3.242 × 10^+4^	1.820 × 10^+4^	**1.794 × 10^+4^**	2.665 × 10^+4^
	Std	3.530 × 10^+4^	2.202 × 10^+4^	1.915 × 10^+4^	**1.842 × 10^+4^**	2.312 × 10^+4^
	Rank	4	5	2	1	3
CEC14	Best	1.977 × 10^+3^	1.925 × 10^+3^	1.834 × 10^+3^	1.695 × 10^+3^	**1.548 × 10^+3^**
	Mean	9.720 × 10^+3^	8.360 × 10^+3^	1.374 × 10^+4^	3.874 × 10^+3^	**3.695 × 10^+3^**
	Std	1.157 × 10^+4^	6.583 × 10^+3^	1.068 × 10^+4^	2.741 × 10^+3^	**2.118 × 10^+3^**
	Rank	3	4	5	2	1
CEC15	Best	2.215 × 10^+3^	1.881 × 10^+3^	1.715 × 10^+3^	1.657 × 10^+3^	**1.524 × 10^+3^**
	Mean	1.489 × 10^+4^	1.353 × 10^+4^	**9.104 × 10^+3^**	1.002 × 10^+4^	1.418 × 10^+8^
	Std	1.970 × 10^+4^	1.456 × 10^+4^	**9.948 × 10^+3^**	1.095 × 10^+4^	7.764 × 10^+8^
	Rank	5	4	3	2	1
CEC16	Best	1.910 × 10^+3^	1.977 × 10^+3^	1.883 × 10^+3^	1.871 × 10^+3^	**1.746 × 10^+3^**
	Mean	2.607 × 10^+3^	2.619 × 10^+3^	**2.449 × 10^+3^**	2.620 × 10^+3^	2.882 × 10^+3^
	Std	**2.702 × 10^+2^**	3.939 × 10^+2^	2.850 × 10^+2^	3.874 × 10^+2^	2.691 × 10^+3^
	Rank	3	4	1	5	2
CEC17	Best	1.915 × 10^+3^	1.800 × 10^+3^	1.741 × 10^+3^	1.748 × 10^+3^	**1.737 × 10^+3^**
	Mean	2.103 × 10^+3^	2.158 × 10^+3^	**1.991 × 10^+3^**	2.090 × 10^+3^	2.043 × 10^+3^
	Std	**1.466 × 10^+2^**	2.355 × 10^+2^	1.712 × 10^+2^	2.031 × 10^+2^	1.914 × 10^+2^
	Rank	4	5	1	3	2
CEC18	Best	2.251 × 10^+4^	3.658 × 10^+4^	4.196 × 10^+4^	**7.205 × 10^+3^**	1.199 × 10^+4^
	Mean	1.818 × 10^+5^	2.476 × 10^+5^	2.789 × 10^+5^	8.728 × 10^+4^	**8.309 × 10^+4^**
	Std	1.752 × 10^+5^	2.327 × 10^+5^	2.479 × 10^+5^	7.751 × 10^+4^	**5.484 × 10^+4^**
	Rank	3	4	5	1	2
CEC19	Best	2.089 × 10^+3^	2.029 × 10^+3^	1.933 × 10^+3^	1.933 × 10^+3^	**1.924 × 10^+3^**
	Mean	1.143 × 10^+4^	1.034 × 10^+4^	1.095 × 10^+4^	1.121 × 10^+4^	**9.254 × 10^+3^**
	Std	1.408 × 10^+4^	1.475 × 10^+4^	1.398 × 10^+4^	1.132 × 10^+4^	**1.102 × 10^+4^**
	Rank	4	2	1	5	3
CEC20	Best	2.125 × 10^+3^	2.205 × 10^+3^	2.140 × 10^+3^	2.057 × 10^+3^	**2.045 × 10^+3^**
	Mean	2.464 × 10^+3^	2.503 × 10^+3^	2.491 × 10^+3^	**2.406 × 10^+3^**	2.479 × 10^+3^
	Std	2.135 × 10^+2^	**1.729 × 10^+2^**	2.067 × 10^+2^	2.249 × 10^+2^	2.277 × 10^+2^
	Rank	2	5	3	1	4
CEC21	Best	2.345 × 10^+3^	**2.200 × 10^+3^**	2.327 × 10^+3^	2.332 × 10^+3^	2.328 × 10^+3^
	Mean	2.399 × 10^+3^	2.388 × 10^+3^	2.357 × 10^+3^	2.359 × 10^+3^	**2.351 × 10^+3^**
	Std	3.286 × 10^+1^	4.583 × 10^+1^	1.912 × 10^+1^	1.513 × 10^+1^	**1.364 × 10^+1^**
	Rank	5	4	2	3	1
CEC22	Best	**2.300 × 10^+3^**	**2.300 × 10^+3^**	**2.300 × 10^+3^**	**2.300 × 10^+3^**	**2.300 × 10^+3^**
	Mean	**3.404 × 10^+3^**	3.662 × 10^+3^	3.992 × 10^+3^	3.616 × 10^+3^	4.077 × 10^+3^
	Std	**2.057 × 10^+3^**	2.193 × 10^+3^	2.482 × 10^+3^	2.093 × 10^+3^	2.276 × 10^+3^
	Rank	3	4	5	1	2
CEC23	Best	2.701 × 10^+3^	2.706 × 10^+3^	2.691 × 10^+3^	**2.400 × 10^+3^**	2.679 × 10^+3^
	Mean	2.760 × 10^+3^	2.776 × 10^+3^	2.720 × 10^+3^	2.718 × 10^+3^	**2.710 × 10^+3^**
	Std	3.013 × 10^+1^	5.079 × 10^+1^	1.871 × 10^+1^	6.572 × 10^+1^	**1.696 × 10^+1^**
	Rank	4	5	2	3	1
CEC24	Best	2.893 × 10^+3^	2.870 × 10^+3^	**2.848 × 10^+3^**	2.853 × 10^+3^	2.859 × 10^+3^
	Mean	2.960 × 10^+3^	3.009 × 10^+3^	**2.875 × 10^+3^**	2.923 × 10^+3^	2.884 × 10^+3^
	Std	8.248 × 10^+1^	1.994 × 10^+2^	**1.511 × 10^+1^**	9.380 × 10^+1^	1.750 × 10^+1^
	Rank	4	5	1	3	2
CEC25	Best	2.884 × 10^+3^	2.884 × 10^+3^	2.884 × 10^+3^	2.884 × 10^+3^	**2.883 × 10^+3^**
	Mean	2.894 × 10^+3^	2.891 × 10^+3^	2.888 × 10^+3^	**2.887 × 10^+3^**	3.114 × 10^+3^
	Std	1.386 × 10^+1^	1.241 × 10^+1^	1.034 × 10^+1^	**4.884 × 10^+0^**	1.244 × 10^+3^
	Rank	5	4	1	2	3
CEC26	Best	2.800 × 10^+3^	2.800 × 10^+3^	2.900 × 10^+3^	2.900 × 10^+3^	2.900 × 10^+3^
	Mean	4.662 × 10^+3^	4.275 × 10^+3^	4.200 × 10^+3^	4.450 × 10^+3^	**4.174 × 10^+3^**
	Std	6.194 × 10^+2^	9.792 × 10^+2^	3.730 × 10^+2^	4.136 × 10^+2^	**2.791 × 10^+2^**
	Rank	5	4	1	3	2
CEC27	Best	3.210 × 10^+3^	3.217 × 10^+3^	**3.200 × 10^+3^**	3.204 × 10^+3^	3.210 × 10^+3^
	Mean	3.309 × 10^+3^	3.403 × 10^+3^	**3.250 × 10^+3^**	3.269 × 10^+3^	3.355 × 10^+3^
	Std	1.130 × 10^+2^	3.264 × 10^+2^	**3.441 × 10^+1^**	5.323 × 10^+1^	6.116 × 10^+2^
	Rank	4	5	2	3	1
CEC28	Best	**3.100 × 10^+3^**	3.162 × 10^+3^	3.123 × 10^+3^	3.102 × 10^+3^	3.142 × 10^+3^
	Mean	3.367 × 10^+3^	3.224 × 10^+3^	3.316 × 10^+3^	**3.200 × 10^+3^**	3.215 × 10^+3^
	Std	8.128 × 10^+2^	3.479 × 10^+1^	5.677 × 10^+2^	3.901 × 10^+1^	**3.406 × 10^+1^**
	Rank	5	4	3	1	2
CEC29	Best	3.405 × 10^+3^	3.513 × 10^+3^	3.439 × 10^+3^	**3.359 × 10^+3^**	3.361 × 10^+3^
	Mean	4.128 × 10^+3^	4.169 × 10^+3^	3.917 × 10^+3^	3.927 × 10^+3^	**3.790 × 10^+3^**
	Std	4.674 × 10^+2^	6.253 × 10^+2^	2.859 × 10^+2^	4.243 × 10^+2^	**2.776 × 10^+2^**
	Rank	5	4	2	3	1
CEC30	Best	6.976 × 10^+3^	6.156 × 10^+3^	5.882 × 10^+3^	5.577 × 10^+3^	**5.074 × 10^+3^**
	Mean	3.282 × 10^+5^	2.679 × 10^+5^	2.896 × 10^+4^	1.207 × 10^+4^	**1.042 × 10^+4^**
	Std	1.449 × 10^+6^	1.213 × 10^+6^	7.614 × 10^+4^	5.092 × 10^+3^	**4.039 × 10^+3^**
	Rank	5	4	3	2	1
Mean Rank		3.93	4.17	2.83	2.34	1.72
Final Ranking		4	5	3	2	1

**Table 7:** Parameters setting.

Algorithms	Parameters	Setting Value
SSA	Leader position update probability	$c_{3}=0.5$
HHO	Sensitive parameter	α=5
	β	1.5
CPO	Number of cycles	T=2
	Convergence rate	α=0.2
	Trade-off factor	$T_{f}=0.8$

**Table 8:** Mountain model parameters and threat model parameters.

Scene	Mountain Center (*x_i_*, *y_i_*)	Mountain Slope (*a_i_*, *b_i_*)	Mountain Height *h_i_*	Threat Center (*x_k_*, *y_k_*)
1	(27, 26); (19, 58): (55, 59); (60, 33); (46, 78); (79, 55)	(9, 9); (8, 8), (8, 8); (9, 9); (8, 8); (8, 8)	1.7; 2; 1.7; 1.6; 1.8; 1.7	(45, 41); (75, 71)
2	(21, 23); (19, 41); (39, 38); (48, 54); (43, 21); (46, 78); (77, 49); (74, 79); (71, 24)	(5, 5); (6, 6); (6, 6); (6, 6); (7, 7); (7, 7); (7, 7); (6, 6); (7, 7)	1.6; 1.8; 2; 1.7; 1.5; 1.8; 1.5; 1.4; 1.6	(58, 30); (38, 59); (63, 65)
3	(19, 21); (19, 41); (30, 85); (42, 39); (60, 28); (52, 52); (59, 14); (57, 68); (45, 81); (80, 19); (81, 66); (15, 75)	(5, 5); (6, 6); (5, 5); (5, 5); (6, 6); (5, 5); (6, 6); (6, 6); (5, 5); (7, 7); (6, 6); (6, 6)	1.6; 1.8; 1.7; 2; 1.6; 1.7; 1.5; 1.7; 1.8; 1.5; 1.4; 1.6	(60, 30); (45, 75); (20, 40); (80, 70)

**Table 9:** Path planning results.

Scene	Index	PSO	SSA	HHO	DBO	CPO	HBA	SaCHBA_PDN	LRMHBA
1	Best	44.470	47.065	48.700	44.665	45.539	42.052	42.209	**41.890**
	Mean	49.251	48.614	2372.037	52.713	47.734	49.933	46.382	**43.630**
	Std	2.072	**0.980**	4280.114	6.871	1.252	6.007	1.862	2.069
	Friedman	6	4	8	7	3	5	2	1
2	Best	42.954	45.692	48.199	44.742	48.211	**42.542**	44.494	42.644
	Mean	377.820	47.287	5026.148	714.320	53.129	47.192	47.855	**45.446**
	Std	1817.344	**1.263**	5058.883	2524.131	2.277	3.568	3.016	2.327
	Friedman	2	4	8	6	7	3	5	1
3	Best	41.679	50.702	51.880	42.985	54.234	45.592	**39.230**	45.982
	Mean	721.730	52.307	7016.976	4695.648	65.788	52.980	56.384	**48.253**
	Std	2522.122	**1.213**	4634.542	5046.594	7.544	6.130	6.852	1.587
	Friedman	5	2	8	6	7	3	4	1
Mean Rank		4.33	3.33	8	6.33	5.67	3.67	3.67	1
Ranking		5	2	7	6	5	3	3	1

## Data Availability

The data used to support the findings of this study are included in the article.

## Appendix A

**Table A1:** CEC2017 Test Functions.

	No.	Functions	$F_{i}^{*}=F_{i}(X^{*})$
Unimodal Functions	1	Shifted and Rotated Bent Cigar Function	100
	3	Shifted and Rotated Zakharov Function	200
Simple Multimodal Functions	4	Shifted and Rotated Rosenbrock’s Function	300
	5	Shifted and Rotated Rastrigin’s Function	400
	6	Shifted and Rotated Expanded Scaffer’s F6 Function	500
	7	Shifted and Rotated Lunacek Bi_Rastrigin Function	600
	8	Shifted and Rotated Non-Continuous Rastrigin’s Function	700
	9	Shifted and Rotated Levy Function	800
	10	Shifted and Rotated Schwefel’s Function	900
Hybrid Functions	11	Hybrid Function 1 (N = 3)	1000
	12	Hybrid Function 2(N = 3)	1100
	13	Hybrid Function 3 (N = 3)	1200
	14	Hybrid Function 4 (N = 4)	1300
	15	Hybrid Function 5 (N = 4)	1400
	16	Hybrid Function 6 (N = 4)	1500
	17	Hybrid Function 6 (N = 5)	1600
	18	Hybrid Function 6 (N = 5)	1700
	19	Hybrid Function 6 (N = 5)	1800
	20	Hybrid Function 6 (N = 6)	1900
Composition Functions	21	Composition Function 1 (N = 3)	2000
	22	Composition Function 2 (N = 3)	2100
	23	Composition Function 3 (N = 4)	2200
	24	Composition Function 4 (N = 4)	2300
	25	Composition Function 5 (N = 5)	2400
	26	Composition Function 6 (N = 5)	2500
	27	Composition Function 7 (N = 6)	2600
	28	Composition Function 8 (N = 6)	2700
	29	Composition Function 9 (N = 3)	2800
	30	Composition Function 10 (N = 3)	2900
$\mathrm{Search}\;\mathrm{Range}:\;{\left[-100,100\right]}^{D}$			
